# Comparative Genomics and Physiological Investigations Supported Multifaceted Plant Growth-Promoting Activities in Two Hypericum perforatum L.-Associated Plant Growth-Promoting Rhizobacteria for Microbe-Assisted Cultivation

**DOI:** 10.1128/spectrum.00607-23

**Published:** 2023-05-18

**Authors:** Yog Raj, Anil Kumar, Sareeka Kumari, Rakshak Kumar, Rakesh Kumar

**Affiliations:** a Agrotechnology Division, CSIR-Institute of Himalayan Bioresource Technology, Palampur, India; b High Altitude Microbiology Laboratory, Biotechnology Division, CSIR-Institute of Himalayan Bioresource Technology, Palampur, India; c Academy of Scientific and Innovative Research (AcSIR), Ghaziabad, India; University of Minnesota Twin Cities

**Keywords:** genome sequencing, comparative genomics, *Hypericum perforatum*, microbe-assisted cultivation, plant growth-promoting rhizobacteria, rhizomicrobiome

## Abstract

Plants are no longer considered standalone entities; instead, they harbor a diverse community of plant growth-promoting rhizobacteria (PGPR) that aid them in nutrient acquisition and can also deliver resilience. Host plants recognize PGPR in a strain-specific manner; therefore, introducing untargeted PGPR might produce unsatisfactory crop yields. Consequently, to develop a microbe-assisted Hypericum perforatum L. cultivation technique, 31 rhizobacteria were isolated from the plant’s high-altitude Indian western Himalayan natural habitat and *in vitro* characterized for multiple plant growth-promoting attributes. Among 31 rhizobacterial isolates, 26 produced 0.59 to 85.29 μg mL^−1^ indole-3-acetic acid and solubilized 15.77 to 71.43 μg mL^−1^ inorganic phosphate; 21 produced 63.12 to 99.92% siderophore units, and 15 exhibited 103.60 to 1,296.42 nmol α-ketobutyrate mg^−1^ protein h^−1^ 1-aminocyclopropane-1-carboxylate deaminase (ACCD) activity. Based on superior plant growth-promoting attributes, eight statistically significant multifarious PGPR were further evaluated for an *in planta* plant growth-promotion assay under poly greenhouse conditions. Plants treated with Kosakonia cowanii HypNH10 and Rahnella variigena HypNH18 showed, by significant amounts, the highest photosynthetic pigments and performance, eventually leading to the highest biomass accumulation. Comparative genome analysis and comprehensive genome mining unraveled their unique genetic features, such as adaptation to the host plant’s immune system and specialized metabolites. Moreover, the strains harbor several functional genes regulating direct and indirect plant growth-promotion mechanisms through nutrient acquisition, phytohormone production, and stress alleviation. In essence, the current study endorsed strains HypNH10 and HypNH18 as cogent candidates for microbe-assisted *H. perforatum* cultivation by highlighting their exclusive genomic signatures, which suggest their unison, compatibility, and multifaceted beneficial interactions with their host and support the excellent plant growth-promotion performance observed in the greenhouse trial.

**IMPORTANCE** *Hypericum perforatum* L. (St. John’s wort) herbal preparations are among the top-selling products to treat depression worldwide. A significant portion of the overall *Hypericum* supply is sourced through wild collection, prompting a rapid decline in their natural stands. Crop cultivation seems lucrative, although cultivable land and its existing rhizomicrobiome are well suited for traditional crops, and its sudden introduction can create soil microbiome dysbiosis. Also, the conventional plant domestication procedures with increased reliance on agrochemicals can reduce the diversity of the associated rhizomicrobiome and plants’ ability to interact with plant growth-promoting microorganisms, leading to unsatisfactory crop production alongside harmful environmental effects. Cultivating *H. perforatum* with crop-associated beneficial rhizobacteria can reconcile such concerns. Based on a combinatorial *in vitro*, *in vivo* plant growth-promotion assay and *in silico* prediction of plant growth-promoting traits, here we recommend two *H. perforatum*-associated PGPR, Kosakonia cowanii HypNH10 and *Rahnella variigena* HypNH18, to extrapolate as functional bioinoculants for *H. perforatum* sustainable cultivation.

## INTRODUCTION

Hypericum perforatum L. (Hypericaceae), known as St. John’s wort (SJW), is a perennial, yellow-flowering shrubby herb (1). The herb is commonly distributed across Asia, Australia, Europe, and North Africa, and North and South America (2). In India, the herb grows naturally between 1,000 to 3,000 m above mean sea level (amsl), ranging from hills of central parts of the country up to the higher altitudes in the Greater Himalaya (3). The herb usually prefers warm sunny locations such as meadows, forests, grasslands, glades, and fields. The densely leaved dried aerial parts of flower-bearing stems devoid of any woody part (*hyperici herba*) are one of the top-selling preparations (4) used worldwide to treat major depressive disorders (5) or even consumed as a dietary supplement, with average annual sales of over US $6 billion (6). *Hyperici herba* contains hyperforin (phloroglucinol) in translucent glands and hypericin and pseudohypericin (naphtodianthrones) in dark glands (7). Hyperforin is responsible for antidepressant activity (8), while hypericin has been identified as a promising anticancer drug and a prospective therapy for Alzheimer’s disease (9).

The crop is primarily produced in Belarus, Germany, Italy, Poland, Romania, Switzerland, and Siberia. However, harvesting of wild plants accounts for a significant portion of the overall *Hypericum* supply, resulting in an unacceptably rapid decline in their natural stands (10, 11). Deliveries of raw materials from natural habitat to international markets are furnished by almost all nations, including China, Serbia, and the United States (12). It appears that cultivating *H. perforatum* could be a great source of income for Indian farmers also, although in India, there are no standardized agrotechnologies developed yet for its cultivation. The slow crop growth, low production, and nonstandardized quality are significant constraints impeding its cultivation (13). Moreover, cultivable land and its existing rhizomicrobiome are well suited for traditional crops. Therefore, a sudden introduction of medicinal plants, including *H. perforatum*, can interrupt the soil microbiome’s ecological stability and create negative plant-soil feedback by releasing certain autotoxins/antimicrobial compounds through their root exudates (14–16). Also, the conventional domestication procedure for *H. perforatum* can reduce the diversity of the associated rhizomicrobial population together with the loss of the plant’s ability to interact with plant growth-promoting microorganisms (17), eventually leading to unsatisfactory crop production (18). Apart from this, introducing nonnative and untargeted plant growth-promoting microorganisms may impact other aspects of ecosystem functioning, such as changes in the resident microbiome, nutrient cycling, and organic matter persistence (19).

Considering that the *H. perforatum* plants were growing well in their natural stands without agrotechnological management, it was speculated that crop cultivation could be improvised by harnessing the crop-associated indigenous plant growth-promoting rhizobacteria (PGPR) from its natural habitat. The plant-associated PGPR can substantially augment the growth and development of the host plant holistically, through either direct or indirect plant growth-promotion mechanisms. Moreover, crop-specific native PGPR could perform better than nonnative ones, as they adapt better to their specific host plant/natural conditions and have superior fitness, survival rates, and unison with the host plant (20, 21). Therefore, it is critical to isolate them from the target plants’ rhizosphere (22). Furthermore, crop-specific, native PGPR can improve plants’ efficacy by providing somewhat similar conditions as in the location from whence they were isolated (23). Concomitantly, they can also provide resilience to plants to confront various stressors by accelerating plants’ genomic capacity (16, 24). Thereby, considering the skyrocketing demand for *H. perforatum* and to develop innovative and sustainable production techniques, explorations of Indian western Himalayan habitat were made to harness the native crop-specific cultivable PGPR. It was hypothesized that the *H. perforatum* resident rhizomicrobiome, under natural settings, can be a potential source for harnessing efficient PGPR for microbe-assisted *H. perforatum* cultivation in nonnative places. The salient objectives of the current investigation were to (i) harness and identify the cultivable, beneficial rhizobacterial communities associated with *H. perforatum* from its natural habitat in Indian western Himalaya; (ii) *in vitro* characterize multiple plant growth-promoting attributes of harnessed rhizobacterial isolates and evaluate their plant-beneficial potentials on *H. perforatum* agronomical traits such as biomass accumulation, photosynthetic pigments, and performance by *in planta* greenhouse experiments; and (iii) determine the most efficient isolates and decipher their unique genetic features and plant growth-promoting traits (PGPTs) to support their multifaceted and extensive plant growth-promotion performance.

## RESULTS

### Site description of rhizospheric soil collection, its physicochemical characteristics, and description of rhizobacterial isolates.

**FIG 1:**
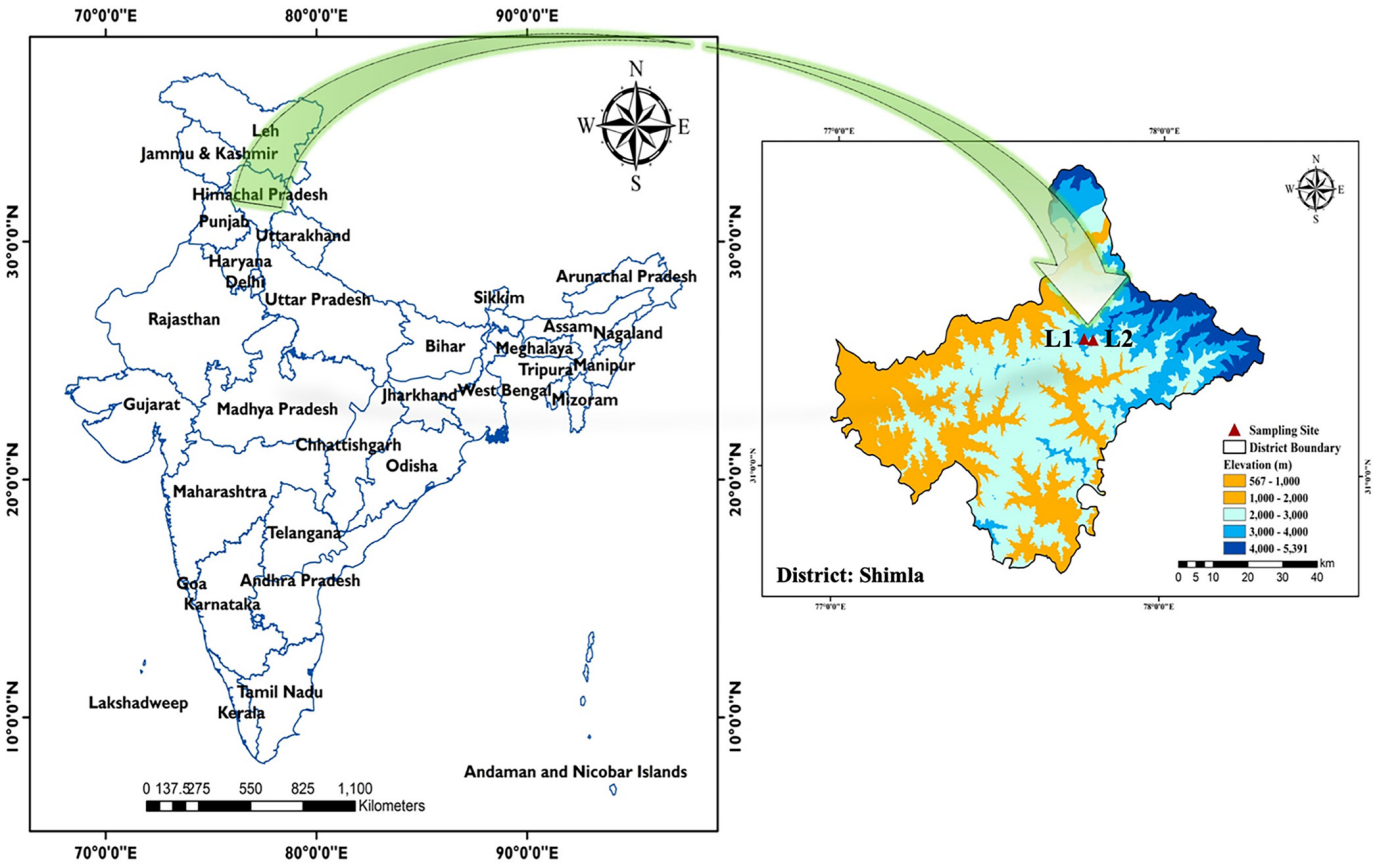
Site description of *H. perforatum* rhizospheric soil collection in Indian western Himalaya. L1, sloppy meadows of Mural Danda peak (altitude, 2,912 m amsl; latitude, 31°18′39″N; longitude, 77°45′40″E); L2, forest fields of Dhumrera village (altitude, 2,679 m amsl; latitude, 31°18′31″N; longitude, 77°47′19″E), Shimla, Himachal Pradesh, India.

Rhizospheric soil samples were collected from the naturally growing *H. perforatum* at the flowering stage (mid-June) from the sloppy meadows of Mural Danda peak (altitude, 2,912 m amsl; latitude, 31°18′39″N; longitude, 77°45′40″E) and forest fields of Dhumrera village (altitude, 2,679 m amsl; latitude, 31°18′31″N; longitude, 77°47′19″E), Shimla, Himachal Pradesh, India (Fig. 1). The soil was characterized as acidic in pH (5.0), fair in organic carbon (0.91%), moderate in available nitrogen (N) (181.3 kg ha^−1^), low in available phosphorous (P_2_O_5_) (8.9 kg ha^−1^), and fair in available potassium (K) (291.3 kg ha^−1^). In total, 31 presumptive rhizobacterial colonies showing a clear halozone in Pikovskaya’s and Ashby’s glucose agar media with unique morphotypes (see Table S1 in the supplemental material) were further restreaked onto a nutrient agar medium to acquire a pure culture.

### *In vitro* plant growth-promoting traits, screening of putative isolates for *in planta* plant growth-promotion assay, and phylogenetic inferences.

**FIG 2:**
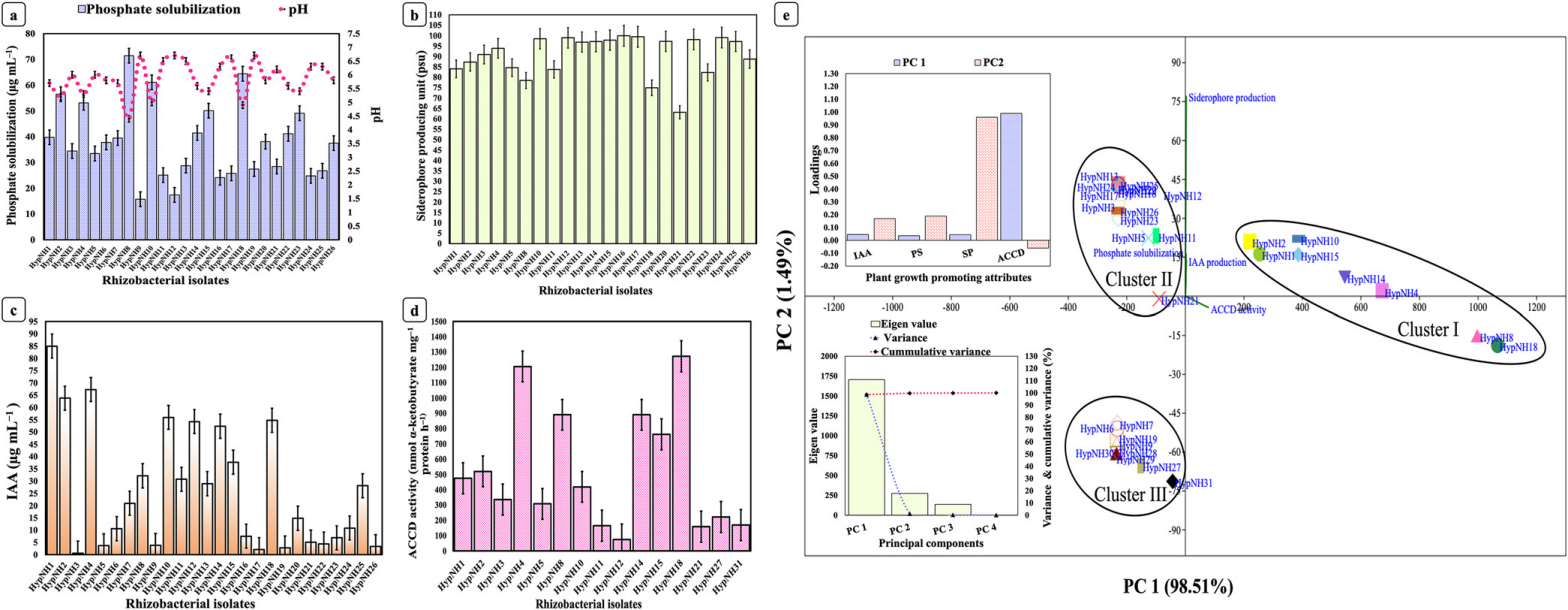
*In vitro* plant growth-promoting attributes of isolated rhizobacteria and screening of putative PGPR. (a) Phosphate solubilization. (b) Siderophore production. (c) Indole-3-acetic acid (IAA) production. (d) 1-Aminocyclopropane-1-carboxylate deaminase (ACCD) activity. (e) Multivariate principal-component analysis (PCA) showing eight statistically significant putative PGPR (cluster I). Results are presented as the means of three replications (*n* = 3) ± the standard error (SE); isolates showing no activity were omitted.

**FIG 3:**
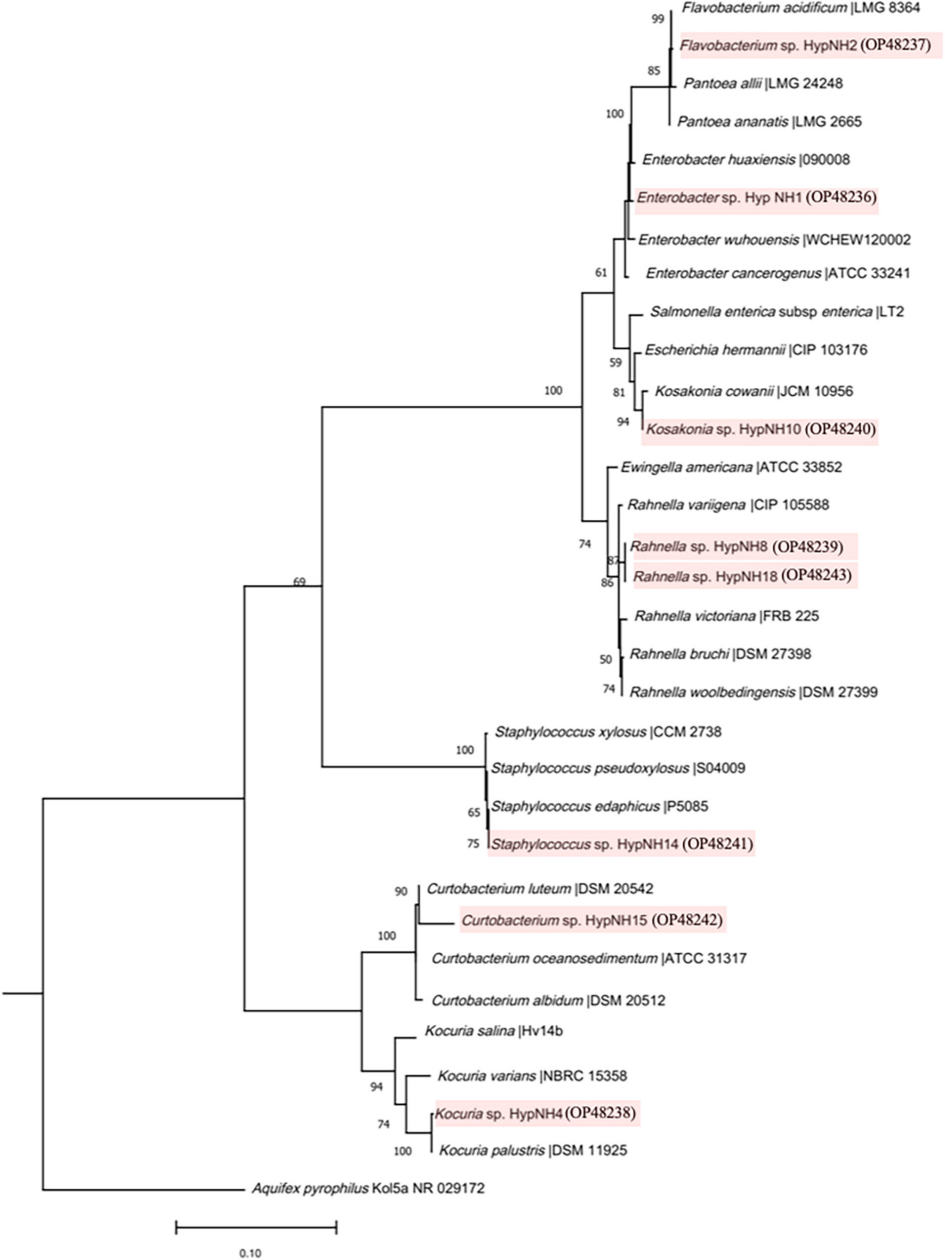
Maximum likelihood based 16S rRNA gene phylogenetic analysis and evolutionary relationship among eight PGPR used for the *in planta* plant growth promotion assay. Closely related type strains were obtained from the EzBioCloud database, and the phylogenetic tree was generated using raxmlGUI 2.0 with the GTR model. Aquifex pyrophilus Kol5aT was taken as the outgroup organism. The value at the nodes represents the bootstrap support values; the accession numbers of putative PGPR strains are shown in parenthesis.

The qualitative estimation of phosphate solubilization capacity revealed that out of 31 rhizobacterial isolates, 20 (64.51%) were capable of phosphate solubilization (Fig. S1). The phosphate solubilizing index (PSI) ranged from 1.45 to 3.24 (Table S2). Additionally, in a quantitative assay, 26 (83.87%) isolates solubilized inorganic phosphate ranging from 15.77 to 71.43 μg mL^−1^ (Fig. 2a; Fig. S2). Accounting for nitrogen (N) fixation ability, 14 isolates (45.16%) exhibited atmospheric free di-nitrogen (N_2_) fixation activity. The N fixation index (NFI) ranged from 1.56 to 3.34 (Fig. S3; Table S3), while potassium (K) mobilization was demonstrated by 13 isolates (41.93%), and the K mobilization index (KMI) ranged from 1.50 to 3.50 (Fig. S4; Table S4). A total of 22 (70.97%) isolates were positive for siderophore production, ranging from 63.12 to 99.92 practical salinity units (PSU) (Fig. 2b; Table S5; Fig. S5 and S6). Phytohormone indole-3-acetic acid (IAA) production ranging from 0.59 to 84.89 μg mL^−1^ was exhibited by 26 isolates (83.87%) (Fig. 2c; Fig. S7), while 1-aminocyclopropane-1-carboxylate deaminase (ACCD) activity ranged from 103.60 to 1,296.42 nmol α-ketobutyrate mg^−1^ protein h^−1^ (Fig. 2d; Fig. S8) and was demonstrated by 15 isolates (48.38%). Eight statistically significant PGPR strains were screened based on the multiple plant growth-promoting attributes (Fig. 2a to d; Table S6) and multivariate principal-component analysis (PCA). The strains were identified up to the species level based on 16S rRNA gene high similarity (>98%) with their closest match and apparent phylogenetic clustering in the same branch. Strains were affiliated with diverse taxa, *viz.*, Enterobacter huaxiensis HypNH1, Flavobacterium acidificum HypNH2, Kocuria palustris HypNH4, Rahnella woolbedingensis HypNH8, Kosakonia cowanii HypNH10, Staphylococcus edaphicus HypNH14, Curtobacterium albidum HypNH15, and Rahnella variigena HypNH18 (Fig. 3, Table S7). These putative PGPR strains were further evaluated individually for *in planta* plant growth-promoting performance in a greenhouse pot experiment (Table S8).

### Efficacy of bioinoculants in an *in planta* plant growth-promotion assay under poly greenhouse conditions.

A poly greenhouse pot experiment was performed to evaluate the efficacy of the eight best PGPR strains. Each pot was planted with a single *H. perforatum* 60-day-old seedling (Fig. S9), and nine individual treatments consisting of eight putative PGPR strains and one uninoculated control (mock) were set up (Table S8). The inoculation of the PGPR strains was done by suspending bacterial cells in sterile normal saline (0.9%) and adjusting their final population to 1 × 10^8^ CFU mL^−1^ at two time intervals: (i) the first dose at transplanting and (ii) the second dose (booster) 15 days after the first inoculation.

### Crop growth and biomass accumulation.

**FIG 4:**
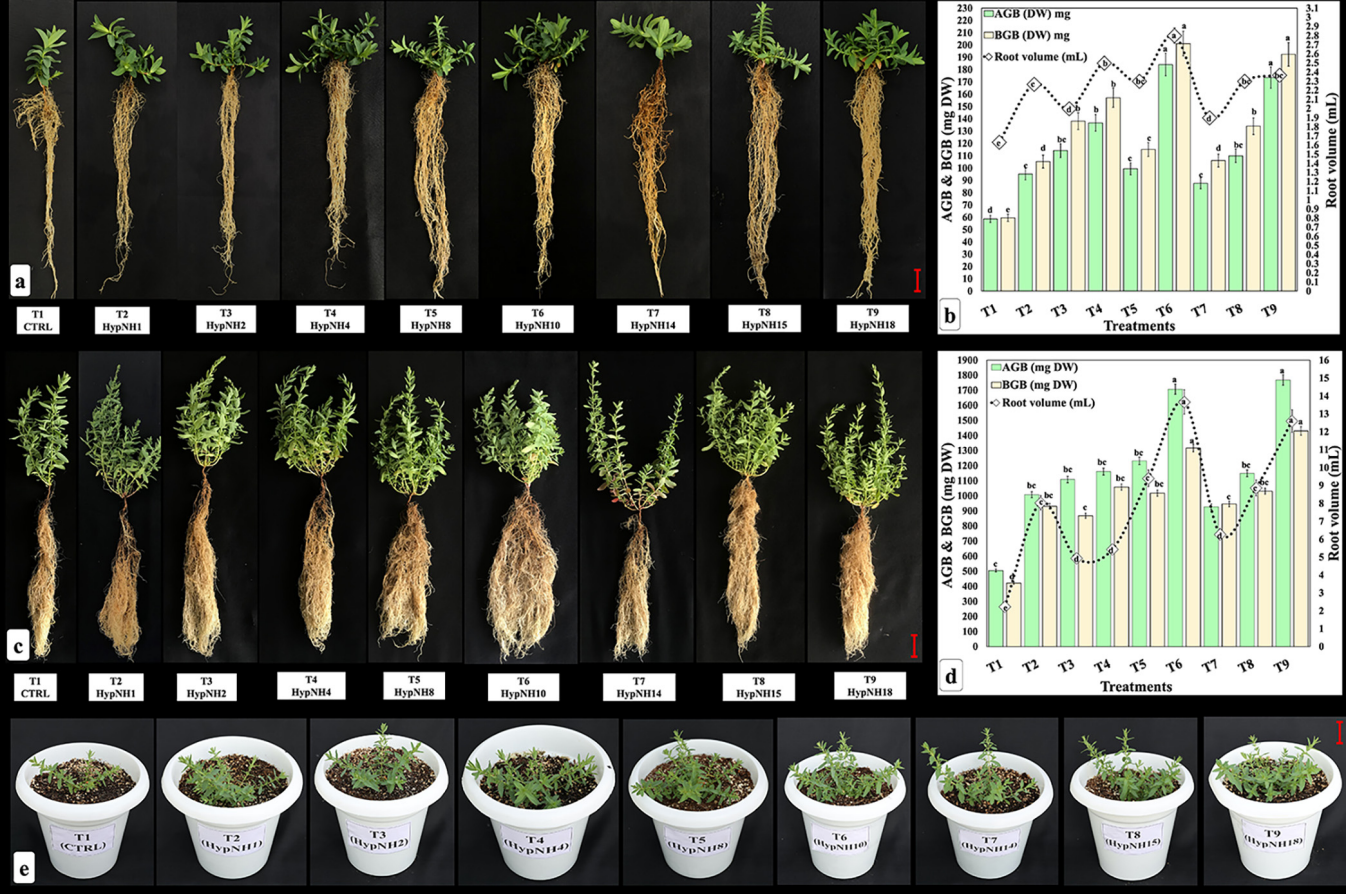
Effect of PGPR treatment on *H. perforatum* agronomical attributes 45 and 90 days after booster treatment. (a and b) Effect on plant growth- and biomass accumulation-related attributes 45 days after booster treatment. (c and d) Effect on plant growth- and biomass accumulation-related attributes 90 days after booster treatment. (e) Overview of the treatment effect. Results are represented as the means of three replications (*n* = 3); same columns with different letters are significantly different at a *P* value of <0.05. Bars = 1 cm; AGB, above dry ground biomass (mg DW); BGB, below dry ground biomass (mg DW); DW, dry weight; T1, control (uninoculated); T2, treatment with *Enterobacter huaxiensis* HypNH1; T3, treatment with Flavobacterium acidificum HypNH2; T4, treatment with Kocuria palustris HypNH4; T5, treatment with *Rahnella woolbedingensis* HypNH8; T6, treatment with Kosakonia cowanii HypNH10; T7, treatment with *Staphylococcus edaphicus* HypNH14; T8, treatment with Curtobacterium albidum HypNH15; T9, treatment with Rahnella variigena HypNH18.

*H. perforatum* plants were destructively harvested at two time points, i.e., 45 and 90 days after booster treatment, to assess any change in plant growth and biomass accumulation. Excluding branches, all other crop agronomical attributes, such as shoot length, root length, above-ground dry biomass (AGB), below-ground dry biomass (BGB), and root volume were significantly (*P* < 0.05) influenced by the PGPR inoculation compared with the uninoculated control groups (T1) at 45 days after booster treatment (Fig. 4a and b). The maximum AGB (184.17 mg dry weight [DW]) and BGB (201.27 mg DW) were recorded for T6 compared the other treatments but remained statistically at par with T9 (Fig. 4a and b). Likewise, at 90 days after booster treatment (DABT), the highest AGB (1,768.03 mg DW) was reported for T9, which was statistically at par with T6 (1,749.33 mg DW) (Fig. 4c and d). Treatment T9 had the significantly highest BGB (1,430.50 mg DW) compared to the other treatments but remained at par with T6 (1,317.33 mg DW). Likewise, the highest root volume was reported for T6 (13.67 mL), which was at par with T9 (12.60 mL) compared with the uninoculated control (T1) and the rest of the bioinoculants.

### Leaf photosynthetic pigments, chlorophyll fluorescence, and gaseous exchange.

**FIG 5:**
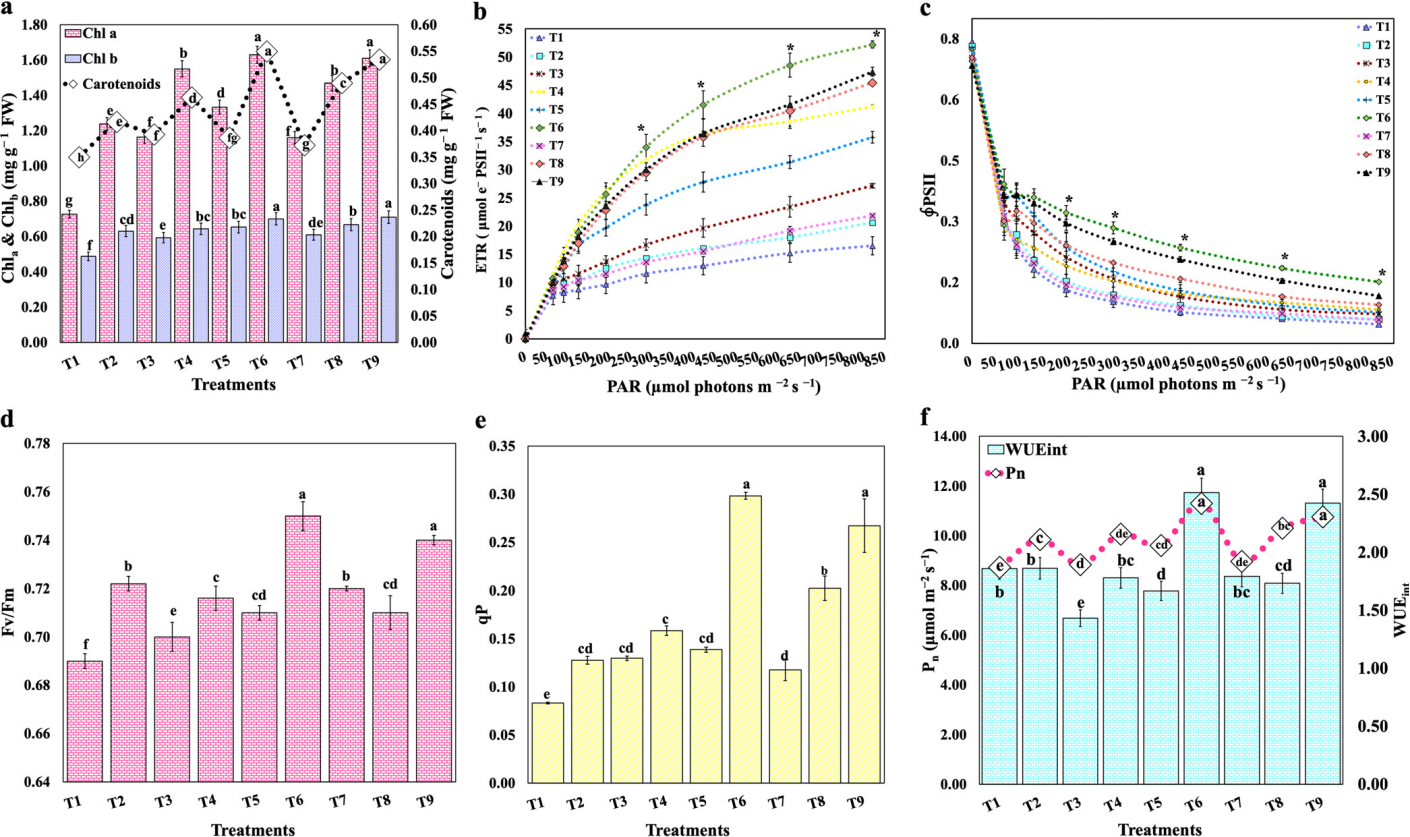
Effect of PGPR treatments on *H. perforatum* physiological performance 90 days after booster treatment. (a) Effect on leaf photosynthetic pigments. (b to e) Effect on leaf chlorophyll florescence related attributes such as ETR (b), ΦPSII (c), F_v_/F_m_ (d), and qP (e). (f) Effect on leaf gaseous exchange-related attributes. Results are represented as the means of three replications (*n* = 3); same columns with different letters are significantly different at a *P* value of <0.05; Chl a, chlorophyll a; Chl b, chlorophyll b; FW, fresh weight; ETR, electron transport rate; ΦPSII, photosystem II operating efficiency; F_v_/F_m_, maximum photochemical quantum efficiency of photosystem II; qP, photochemical quenching; P_n_, net photosynthetic rate (μmol m^−2^ s^−1^); WUE_int_, internal water use efficiency; T1, control (uninoculated); T2, treatment with *Enterobacter huaxiensis* HypNH1; T3, treatment with Flavobacterium acidificum HypNH2; T4, treatment with Kocuria palustris HypNH4; T5, treatment with *Rahnella woolbedingensis* HypNH8; T6, treatment with Kosakonia cowanii HypNH10; T7, treatment with *Staphylococcus edaphicus* HypNH14; T8, treatment with Curtobacterium albidum HypNH15; T9, treatment with Rahnella variigena HypNH18.

The photosynthetic pigments, *viz.*, chlorophyll *a* (Chl_a_), chlorophyll *b* (Chl_b_), carotenoids, and chlorophyll florescence, were estimated at 90 days after booster treatment by collecting fully expended healthy leaves. Leaf Chl_a_ content was significantly (*P* < 0.05) highest in T6 (1.63 mg g^−1^ fresh weight [FW]) compared with the uninoculated control (T1) and the rest of the inoculants but remained at par with T9 (1.61 mg g^−1^ FW) (Fig. 5a). In contrast, Chl_b_ was highest in T9 (0.71 mg g^−1^ FW) compared to other treatments; however, it remained at par with T6 (0.70 mg g^−1^ FW). The concentration of carotenoids was highest in T6 (0.55 mg g^−1^ FW), which is statistically at par with T9 (0.51 mg g^−1^ FW). Likewise, plants treated with PGPR showed a significant increase in the electron transport rate (ETR) of photosystem II (PSII) compared with the mock-inoculated control plants. Furthermore, significant (*P* < 0.05) substantial differences between treatments were seen at above 300 μmol photons m^−2^ s^−1^, with all treatments reaching the maximum ETR (T1, 16.53; T2, 20.67; T3, 27.20; T4, 41.23; T5, 35.77; T6, 52.23; T7, 21.87; T8m 45.50; and T9, 47.40) (Fig. 5b) at 820 μmol photons m^−2^ s^−1^. Likewise, from the lowest to highest photosynthetic active radiation (PAR), PSII operational efficiency (ΦPSII) was also significantly higher in inoculated plants than in the mock groups at PAR above 200 μmol photons m^−2^ s^−1^ (Fig. 5c). The PSII’s maximum photochemical quantum yield (maximum variable fluorescence [F_v_]/maximum Chl fluorescence yield [F_m_]) was also highest in T6 (0.75) compared to all other treatments but remained at par with T9 (0.74). In contrast, the F_v_/F_m_ (0.69) was reported for mock-inoculated control groups (Fig. 5d). Likewise, a similar trend was observed in photochemical quenching (qP) (Fig. 5e). The net photosynthetic rate (P_n_) was highest in T6 (11.64 μmol m^−2^ s^−1^) compared to the other treatments but remained statistically at par with T9 (10.76 μmol m^−2^ s^−1^) (Fig. 5f). The maximum internal water use efficiency (WUE_int_) (2.51) was also significantly higher in T6 than in all other treatments and remained statistically at par with T9 (2.42). Overall, in pot experiments, all PGPR strains performed well over mock-inoculated controls; however, the plant treated with *K. cowanii* HypNH10 (T6) and *R. variigena* HypNH18 (T9) performed best among all bioinoculants. They were further selected for generating genome resources to decipher their unique genetic features and plant growth-promoting attributes.

### Genome assembly, phylogenomics, comparative genomics, and functional annotation of unique genes encoding plant growth-promoting traits.

Genomic resources of the best-performing PGPR strains, HypNH10 and HypNH18, were generated, and a whole-genome-based phylogeny using the Type Strain Genome Server showed clustering of strain HypNH10 with its closest relative Kosakonia cowanii JCM10956 (Fig. S10a) and strain HypNH18 with Rahnella variigena CIP105588 (Fig. S10b) and was thus affiliated as *K. cowanii* HypNH10 and *R. variigena* HypNH18. The draft genome of *K. cowanii* strain HypNH10 was 4.72 Mb with a 55.00% G+C content and a total of 4,470 predicted genes, including 4,310 coding genes (Table S9). On the other hand, the genome of *R. variigena* HypNH18 was 5.32 Mb with a 52.15% G+C content and 4,915 total predicted genes; out of those, 4,798 were functional/protein-coding genes (Table S10).

**FIG 6:**
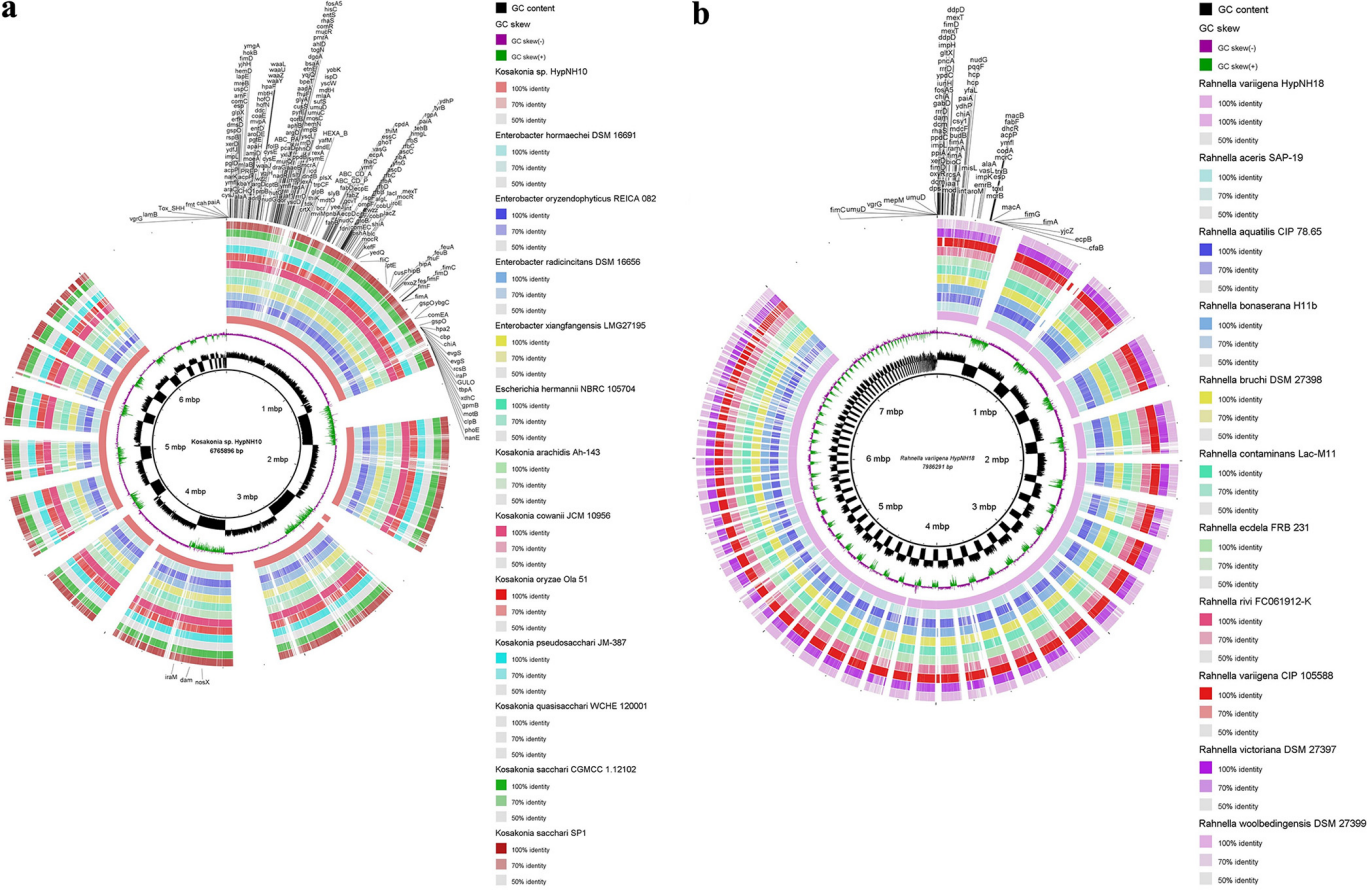
Circular chromosomal maps of two PGPR strains, (a) Kosakonia cowanii HypNH10 and (b) Rahnella variigena HypNH18. Pangenome analysis with closely related strains identified the unique genes present in the query genomes that are highlighted in the outermost circle. The query genome is placed in the innermost circle.

The pan-genome analysis of *K. cowanii* strain HypNH10 with its 12 closely related strains showed a total of 36,506 genes, out of which 27,010 cloud, 191 core, and 9,305 shell genes were scored. The pan-genome analysis of *R. variigena* HypNH18 with its 10 closely related strains showed a total of 21,275 genes, out of which 14,701 were cloud, 5,077 were shell, and 1,497 were core genes (Table S11). The unique genes of *K. cowanii* HypNH10 and *R. variigena* HypNH18 obtained from pangenome analysis were searched for their plant growth-promoting trait (PGPT)-related traits by performing BLASTp analysis against the PGPT protein reference sequence database. The BLASTp analysis revealed that *K. cowanii* HypNH10 harbored 229 unique functional genes encoding diverse PGPT classes such as biofertilization, phytohormone/plant signal production, colonization/plant-derived exudate usage, competitive exclusion, stress resilience, bioremediation, and plant immune response stimulation (Table S12). In the class biofertilization, the strain has two genes (*icd* and *cah*) encoding proteins for photosynthetic carbon dioxide (CO_2_) fixation via reductive citric acid cycling. It also possesses a series of six genes (*phoE*, *gloB*, *hpaF*, *alaA*, *phnO*, and *prpB*) affiliated with phosphate-solubilization/acquisition through the solubilization/transport of phosphate, organic acid metabolism, and succinic acid biosynthesis. Additionally, 10 genes (*iroE*, *tbpA*, *ymfI*, *hemN*, *entS*, *ddc*, *feuB*, *mbt*, *entD*, and *ygjH*) encoding siderophore production/iron acquisition, 3 N acquisition-related genes (*draG*, *fdnI*, and *narK*), 2 K-solubilizing genes (*argD* and *yjhH*), and 1 sulfur (S) assimilation/mineralization encoding gene (*dmsD*) were also present in its chromosome (Fig. 6a).

In terms of host plant colonization, *K. cowanii* HypNH10 harbors host invasion-related and plant cell wall membrane degradation-related genes (*rrrD* and *yeeJ*). Moreover, two more genes governing root colonization through niacin (vitamin B3) biosynthesis (*nadR*), and site-specific recombination (*xerD*) were also predicted in the strain. Furthermore, it also harbored 3 chemotaxis-related (*fliC*, *motB*, and *ppdB*) and a series of 15 surface adhesion/attachment-related genes (*rfbB*, *rfbC*, *rfbD*, *rgpA*, *wzz*, *pglD*, *fimD*, *waaJ*, *waaY*, *waaZ*, *waaU*, *waaL*, *lapE*, *ymgA*, and *arnF*), suggesting its close association with the host plant. In addition to plant-derived amino acids, organic acids, carbohydrates, and nucleoside degradation capabilities, this strain has the unique genetic ability to use/degrade plant-derived specialized metabolites/exudates, such as aromatic/phenolic compounds, phenylalanine, and shikimate. At the same time, the PGPT class phytohormone and plant signaling have genes related to phytohormone (IAA) production via the tryptophan pathway, including plant signaling via the terpenoid-isopentenyl diphosphate pathway. Intriguingly, the strain also harbored three genes (*laf*, *fadA*, and *thiM*) affiliated with plant immune response stimulation by triggering an induced systemic resistance mechanism through plant-associated molecular patterns and effector-triggered immunity. Additionally, 31 unique genes encoding PGPT class competitive exclusion attributable to bacterial fitness, especially for adaption to the host plant immune system, resistance against antimicrobial compounds, adaptive mutation through environment-inducible DNA polymerases, and phage exclusion were also predicted. Accounting for the bacterial secretion system, two genes (*gspO* and *cpdA*) encoding biofilm production, five exopolysaccharides (*exoZ*, *bshA*, *adrB*, *HEXA_B*, and *cysE*), four type II secretion system (T2SS; *comEC*, *hofN*, *hofO*, and *mreB*), four T3SS (*yscL*, *yscD*, *yscW*, and *hpa2*), one T5SS (*esp*), and four T6SS production-related genes (*impB*, *essC*, *vgrG*, and *impL*) were also predicted in the *K. cowanii* HypNH10 genome.

The strain also harbors six genes putatively involved in the alleviation of biotic stressors through fungicidal activities such as chitinolytic activities (*cbpI* and *chiA*), phenazine-1-carboxylic acid biosynthesis (*aroDE*), and toxoflavin metabolism (*toxI*). Bactericidal activities of the strain were putatively regulated by genes for aurachin (*acpP*) and spermidine/putrescine metabolism (*paiA*). Interestingly, a unique herbicide resistance gene (*glpB*) via organophosphate degradation was also predicted in its chromosome. In addition, several genes encoding proteins/enzymes required to neutralize various abiotic stressors such as salinity (*ydfJ*, *rspB*, *argO*, and *folB*), acidity (*evgS*), heat (*clpB*), and oxidative stress via carotenoid (*crtX*) and arylpolyene biosynthesis (*ABCCDP*, *ABCCDA*, *fabB*, *ybgC*, and *acpP*) were also predicted. Additionally, a few bioremediation-related genes involved in heavy metal detoxification (*comR*, *pmrA*, and *feuA*), xenobiotic vinyl chloride degradation (*etnE*), and copper, cobalt, iron, and tellurium resistance were also predicted in the strain.

The strain Rahnella variigena HypNH18 harbored 82 unique PGPT-encoding genes regulating biofertilization, phytohormone/plant signal production, colonization and plant-derived substrate/exudate usage, competitive exclusion, and resilience to biotic and abiotic stressors (Table S13). Accounting for the PGPT class biofertilization, the strain harbored two genes (*pqqF* and *mdcF*) regulating phosphate solubilization/acquisition through the gluconic acid-PQQ pathway and gluconic acid transport, respectively. Additionally, three genes (*macA*, *macB*, and *dps*) encoding the siderophore export system and iron homeostasis-related proteins and one N acquisition-related gene (*eutM*) were also present on its chromosome (Fig. 6b).

In terms of plant colonization, five functional genes dedicated to root colonization through niacin (*pncA*) and biotin (*ymfI* and *bioC*) biosynthesis, root nodule regulation (*nodD*), and site-specific recombinase activity (*xerD*) were also present in strain HypNH18’s chromosome. Moreover, the strain also harbored four chemotaxis-related genes (*fimA*, *fimG*, *sfmH*, and *fimC*) and three surface attachment-related genes (*fimA*, *cfaB*, and *fimD*) that encode its target-oriented movement toward root exudates. Furthermore, it also contained a unique *aroM* gene that encodes the strain’s distinct genetic ability to use/degrade plant-derived metabolites/exudates and even shikimate. The classes phytohormone and plant signaling have functional genes encoding phytohormone (IAA) production via the IAA pathway (*iaaT*), including plant signaling through the production of volatile organic compounds such as acetoin 2,3-butanediol biosynthesis (*budB*) and heme/siroheme biosynthesis (*gltX*).

In addition, 31 functional genes related to competitive exclusion attributable to bacterial fitness, primarily through the type I CRISPR-Cas system (e.g., *csy1*), resistance to plant antimicrobial peptides (e.g., *ppiA*), and adaptive mutation (e.g., *umuD*) through environment-inducible DNA polymerases were also predicted. Accounting for the bacterial secretion system, two biofilm- (*paiA* and *gcvA*), one exopolysaccharide- (*ramA*), one T1SS- (*fimA*), one T4SS- (*ppdC*), and six T6SS-related (*vgrG*, *impL*, *hcp*, *impK*, *vasL*, and *impH*) functional genes were also in strain HypNH18’s genome. The strain also encompasses four genes that regulate biotic stress alleviation through fungicidal activities via chitinolytic activities (e.g., *chiA*) and toxoflavin metabolism (e.g., *toxI*). Accounting for bactericidal action, the strain harbored two genes, *acpP* and *fabF*, encoding proteins for aurachin and volatile fatty acid metabolism, respectively. Additionally, several functional genes having the capacity to neutralize various abiotic stressors, such as osmotic and oxidative stressors, through niacin biosynthesis (*iunH*), thioredoxin/thioesterase activity (*trxB*), and detoxification of per-oxidized compounds (*oxyR*), respectively were also predicted.

### Bacterial secondary metabolite biosynthesis gene cluster analysis.

Genome mining using the antiSMASH v6.0 platform predicted seven putative secondary metabolite biosynthesis gene clusters (BGCs), *viz.*, terpene (BGC-1) producing carotenoids, ladderane (BGC-2), thiopeptide (BGC-3), nonribosomally synthesized peptide synthetase (NRPS) (BGC-4) producing turnerbactin, a triscatecholate siderophore, a redox-cofactor type cluster (BGC-5) producing lankacidin C (an antibacterial compound), and unspecified ribosomally synthesized (BGC-6) producing teicoplanin (antibacterial) and a posttranslationally modified peptide product (RiPP-like) (BGC-7) in *K. cowanii* HypNH10 (Fig. S11a). BGC-2 and BGC-3 did not show similarities to those present in the antiSMASH database. Likewise, five key secondary metabolite BGC regions, *viz.*, an RRE-element-containing cluster (BGC-1) producing lankacidin C and betalectone (BGC-2) with antibacterial activities, siderophore (BGC-3) (desferrioxamine E), hserlactone (BGC-4), a volatile compound homoserine lactone responsible for communication between bacteria and fungi, and aryl polyene (BGC-5), which protect the bacterium from reactive oxygen species were carried by *R. variigena* HypNH18 (Fig. S11b).

## DISCUSSION

Despite knowing about the existence and involvement of rhizospheric microorganisms throughout plant development, during plant domestication, humans have selectively chosen traits related only to yield, flavor, or nutrition. However, they have not focused on the beneficial rhizomicrobiome associated with the plant (25). Medicinal plants harbor a peculiar rhizomicrobial community because of their structurally diverse bioactive specialized metabolites, root exudates, and rhizodeposits which are accountable for the recruitment of microorganisms (26, 27). Furthermore, the host plant recognizes rhizobacteria in a strain-specific manner (28). Therefore, in the current study attempts were made to harness native multifaceted PGPR from the rhizosphere of the target crop (*H. perforatum*) to develop an alternative sustainable production technology for its domestication in the Indian western Himalaya. The rhizospheric soil was collected from the high-altitude *H. perforatum* natural habitat in the Indian western Himalaya (Fig. 1) during the flowering stage, as the bacterial community was speculated to be the most diverse at this stage (29). The enrichment culture approach was adopted to isolate the efficient PGPR to ensure the isolation of underrepresented taxa, as minimal medium would increase the risk of losing underdocumented species (30). Enrichment isolation allowed recovery of 31 native rhizobacteria from *H. perforatum*, and their quantitative plant growth-promoting properties, such as phosphate solubilization, indole-3-acetic acid (IAA), siderophore production, and 1-aminocyclopropane-1-carboxylate deaminase (ACCD) activity, allowed the selection of eight statistically significant multitrait PGPR, which were further used as putative bioinoculums to demonstrate *in vivo* plant growth-promotion activity under poly greenhouse conditions. Our findings reporting multitrait PGPR are in agreement with some previous studies reporting various plant growth-promoting activities in PGPR harnessed from the rhizosphere of medicinal plants (31, 32), as well as from nonmedicinal plants (33, 34).

However, contrary to our current findings of Enterobacter, *Flavobacterium*, *Curtobacterium*, *Kocuria*, Staphylococcus, *Rahnella*, and *Kosakonia* as the dominant PGPR genera in the rhizosphere of naturally occurring *H. perforatum*, a recent study of the bacterial community structural composition in the rhizosphere of *Hypericum* species grown under greenhouse conditions in Halle, Germany (Latitude: 51° 29' 42.23” N; Longitude: 11° 56' 36.56” E) using a culture-independent approach suggested the members of genera *Bacillus*, *Bradyrhizobium*, *Mesorhizobium*, *Streptomyces*, and *Pseudonocardia* as specific taxa associated with *H. perforatum* and Hypericum olympicum (hypericin and hyperforin-producing species). Furthermore, two novel bacterial strains, Hypericibacter terrae gen. nov., sp. nov. and Hypericibacter adhaerens sp. nov. of the *Rhodospirillaceae* family, were also harnessed from the rhizosphere of 1.5-year-old *H. perforatum* plants, although their plant growth-promoting activities were not evaluated (35). This variability in *H. perforatum*’s rhizobacterial composition might be attributable to the disparity in factors such as criteria and methodology of screening, geographical location, soil edaphic properties, vegetation type, and land use history, which drive the deterministic rhizomicrobial community structure (36). In the current investigation, rhizobacterial isolates were harnessed from the *H. perforatum* natural habitats in the high-altitude environment (Fig. 1). This ecosystem includes a variety of stressors, such as excessive UV radiation, desiccation and freezing and subsequent warming. Consequently, bacteria that inhabit high-altitude regions are prone to reactive oxygen species (37). In such environments, bacteria produce many antioxidative enzymes to reduce the damage caused by reactive oxygen species. The genomic information of the two most effective strains, Kosakonia cowanii HypNH10 and Rahnella variigena HypNH18, shows the presence unique genes encoding proteins for oxidative and abiotic stress resilience in their chromosomes (Table S12 and S13), suggesting them as stress-tolerant high-altitude bacteria.

### Inferences of the *in planta* plant growth-promotion experiment.

The increased crop growth as a result of plant growth-promoting rhizobacterial inoculation observed in the current investigation (Fig. 4a to e) could be attributable to the multifaceted PGPTs such as macronutrient mobilization, phytohormone (IAA), and siderophore production, including ACCD activity of the used PGPR (Fig. 2a to e). Similar findings were reported in a recent article demonstrating a significant increment in the growth of fenugreek plants inoculated with native PGPR, Priestia endophytica SK1 isolated from the target crop under salinity stress (38), although they did not explore the genomic evidence. Soil-beneficial microorganisms fasten the process of biogeochemical cycling of essential elements (C, N, P, K, and S) through solubilization and mineralization, leading to enhanced nutrient availability (39). In soil, phosphorous (P) is usually present in water-insoluble forms bonded with Al^3+^, Fe^3+^, and Ca^2+^, making it difficult for plants to absorb. PGPR with phosphate-solubilizing capacity can convert the insoluble forms of P to soluble forms by reducing the soil pH by secreting organic acids or extracellular enzymes and thereby augment the overall P acquisition by plants (40). Similarly, plants acquire nitrogen (N) only in its inorganic forms, as either nitrate (NO^3−^) or ammonium (NH_4_^+^). PGPR can fix atmospheric dinitrogen (N_2_) into ammonium (NH_4_^+^) by N fixation, while soil organic N can be degraded into NH_4_^+^ by ammonification, which can be subsequently oxidized to NO^3–^ by nitrification or converted back to N_2_ by denitrification (41). Likewise, despite the higher abundance of potassium (K) in soil, just 1 to 2% is accessible to plants through its exchangeable forms. Most soil comprises 90 to 98% mineral K, such as biotite, muscovite, orthoclase, and illite, which are inaccessible to the plant. These inaccessible minerals can be solubilized and made available to plants by PGPR, having K-mobilizing properties (42).

Moreover, it is well documented in various studies that the PGPR usually colonize roots, produce biofilms, and increase root length, root branching, lateral roots (43), root surface, root clusters (44), and root nodules by the secretion of auxins, cytokinins, gibberellic acids, siderophores, exopolysaccharides, and organic acids, leading to better nutrient and water acquisition (45, 46). Additionally, they can also alleviate stress by lowering ethylene levels accumulated during exposure to biotic/abiotic stressors through ACCD activity (47). In addition, some indirect mechanisms, *viz.*, antibiosis, competition, and induced systemic resistance, can also be ascribed to the enhanced plant growth observed in the current study (48–50). In the current investigation, PGPR have altered the root architecture, leading to thinner and longer roots with more proliferating peripheral roots (Fig. 4a and c) with more root volume (Fig. 4b and d), which can boost nutrient acquisition by plants, eventually leading to more biomass accumulation (51). Furthermore, biomass allocation depends on soil N availability; PGPR can act as diazotroph bacteria and facilitate biological N_2_ fixation that could influence the plant’s N budget directly by delivering N by atmospheric N fixation or by providing accessible NH_4_^+^-N and NO^3–^-N through N fixation and nitrification, respectively. Moreover, the bacterial exopolysaccharide (EPS) possesses a net negative charge and unique water-holding and cementing properties; as a result, positively charged ions are efficiently sequestered, facilitating increased water and nutrient acquisition across plant roots through biofilms (52) and consequently producing more plant biomass. Additionally, PGPR secretes IAA using its precursor (l-tryptophan) excreted naturally by the plant roots (53) and induces transcriptional changes in auxin response and cell wall differentiation genes, leading to longer roots and higher root biomass accumulation (54). Consequently, in the current investigation, PGPR-inoculated developing seedlings might have received more nutrients from their surroundings and eventually accumulated more biomass than the mock groups. Concomitantly, IAA can help plants in defense response mechanisms and make them resilient to various stressors. Additionally, siderophores produced by the PGPR primarily bind with ferric ions (Fe^3+^) and translocate iron across the plant’s cell membranes with increased mobility and acquisition. In addition, siderophores can perform as molecular signals in response to environmental factors and even can protect plants from phytopathogens by chelating iron in the rhizosphere (55).

### Effect of bioinoculants on plants’ photosynthetic pigments and performance.

According to our results, PGPR inoculation enhanced the concentrations of leaf photosynthetic pigments (Fig. 5a) and eventually increased chlorophyll (Chl) fluorescence (Fig. 5b to e) and net photosynthetic rate (P_n_) (Fig. 5f). This may be attributable to siderophores produced by the PGPR, providing iron and thus augmenting Chl concentration (Fig. 5a), as iron is involved in the formation of Chl precursor (aminolevulinic acid) (56). The increased photosynthetic pigments detected in the current study are in congruence with a recent study reporting a significant augmentation in the Chl pigments after inoculation of single, dual, and a consortium of Pseudomonas rhizophila S211, Halomonas desertis G11, and Oceanobacillus iheyensis E9 in Pelargonium graveolens L’Hér 90-day-old seedlings under semicontrolled conditions (57). The photosystem II (PSII) electron transport rate was also increased in *H. perforatum* leaves after inoculation with PGPR, suggesting extensive oxidization of quinone acceptor (Q_a_) and its efficient consumption in the electron transport chain (ETC) without causing any photo-injury. Additionally, a significant increase was observed in PSII operating efficiency (ΦPSII), a measure of the amount of light captured by the Chl by PSII, which is further used in photochemical reactions. Furthermore, PGPR-inoculated plants had maximum photochemical quantum yield (F_v_/F_m_), which is the highest efficiency at which light received by PSII is used to reduce Q_a_ and is considered a sensitive indicator of plant photosynthetic ability. Increased F_v_/F_m_ values because of PGPR inoculation were also reported in a previous investigation suggesting stress alleviation in Mentha pulegium L. (58). Moreover, the *H. perforatum* plants inoculated with PGPR showed increased photochemical quenching (qP) compared with mock-inoculated plants; qP signifies the fraction of excitons collected by open traps and converted to chemical energy in the PSII reaction center, which contributes to the reduction of O_2_ generation in the PSII antenna (59). Likewise, the PGPR inoculation modified the leaf gaseous exchange by enhancing net photosynthesis (P_n_) and water use efficiency (WUE_int_); therefore, more CO_2_ molecules will be assimilated per H_2_O molecule liberated through stomata. Interestingly, it has been observed that when plants are treated with PGPR, P_n_ improves significantly as a consequence of enhanced nutrient availability and photosynthetic pigments. Also, the inoculation of PGPR increased the total Chl concentration and Chl fluorescence, implying increased light-harvesting and reaction centers. As a result, Q_a_ is highly oxidized, and its excitation energy is used in ETC, resulting in higher ATP and NADPH production, which are further used for C assimilation during the Calvin (C_3_) cycle, eventually improving the crop biomass (60, 61).

Overall, in the *in planta* assay, almost all eight PGPR promoted the growth of *H. perforatum* seedlings under poly greenhouse conditions, suggesting their successive colonization in native agricultural soil. However, two bioinoculants, *K. cowanii* HypNH10, and *R. variigena* HypNH18, performed best, enhancing the maximum root and shoot biomass (Fig. 4a to e). The plausible reason for this might be the significantly higher enhancement in plant physiological performance indexes such as Chl fluorescence, P_n_, and WUE_int_, as these two isolates have blatantly augmented the photosynthetic pigments along with the photosynthetic performance of the crop compared with the mock groups and the rest of the bioinoculants (Fig. 5a to f). It is well known that plants translocate 40 to 60% of photosynthetically fixed carbon into the root zone and release it as root exudates, providing a source of nutrients for rhizobacterial communities (62, 63). Increased photosynthetic performance of plants inoculated with *K. cowanii* HypNH10 and *R. variigena* HypNH18 suggest that the more photosynthates would be assimilated, and plants will release more exudates to harbor a higher abundance of bioinoculants, providing direct accessory benefits to the associated host plant for nutrient acquisition and phytohormone production and eventually producing higher biomass.

### Unique genetic features and plant growth-promoting traits in Kosakonia cowanii HypNH10 and Rahnella variigena HypNH18.

Based on comprehensive genome mining and functional annotation of plant growth-promotion traits (PGPTs) using a novel PLaBAse database and the online tool (PGPT-Pred), the following functional characteristics of *K. cowanii* HypNH10 and *R. variigena* HypNH18 can be proposed. Both of them have unique genes encoding proteins for resistance to plant antimicrobial peptides and plant immunity. Also, the strains harbor several genes encoding proteins required to degrade/use plant-derived substrates/exudates, including specialized metabolites (shikimate and phenylpropanoid). The strains are motile and have effective interactions with their host plant, as they harbor unique genes encoding proteins required for flagellar assembly, root colonization, competitive exclusion, surface attachment, chemotaxis, biofilm production, and in particular, bacterial secretion systems (T1SS, T3SS, T4SS, and T6SS). Chemotaxis- and surface attachment-related genes are considered pivotal for the recruitment and colonization of bacteria in the rhizosphere (64). In addition, bacterial secretion systems play an essential role in out-competing other rhizobacterial strains during root colonization in the host plant; in particular, T1SS, T3SS, and T4SS are known to be crucial for bacterium-plant interaction, while T6SS is critical for competitive plant colonization (65–68). Furthermore, bacterial biofilms can enhance the growth of plants by acting as biocontrol agents which protect them from various infectious microbes by inducing systemic resistance and the secretion of antimicrobial compounds (69). In addition, both strains harbored genes encoding proteins involved in the production of bacterial volatile organic compounds, which were previously reported to improve plant health (70). Also, they carry a remarkably higher set of direct PGPTs, which might impart direct plant benefits via biofertilization, phytohormones, and plant signal production. In addition, both strains also carried several functional genes putatively involved in abiotic stress alleviation through stimulating the plant immune response and biotic stress management via fungicidal (chitinolytic phenazine-1-carboxylic acid biosynthesis and toxoflavin metabolism) and bactericidal (aurachin and spermidine/putrescine metabolism) activities. Furthermore, based on bacterial secondary metabolite biosynthesis gene cluster analysis, both isolates can be proposed as putative biocontrol agents, as they both harbor many secondary metabolite biosynthesis gene clusters producing diverse antibacterial and antifungal compounds (71).

### Conclusions.

The crop-specific, native PGPR can provide new and innovative avenues for augmenting the amount and quality of *H. perforatum* secondary metabolites by either enhancing plant biomass or regulating its biosynthetic pathways. The present work documents the diversity of culturable PGPR associated with *H. perforatum* from the high-altitude Indian western Himalayan habitat for the first time. Current findings also showed the presence of cogent multifarious culturable PGPR associated with *H. perforatum* for its extrapolation as functional bioinoculants for microbe-assisted cultivation. Consequently, this study will significantly contribute to the sustainable cultivation of *H. perforatum* and can assist the biofertilizer industries in developing a putative bioinoculant exclusively for medicinal plants. Furthermore, the current findings have improved our knowledge of the role of native crop-specific PGPR strains in enhancing host plants’ physiological processes, such as leaf chlorophyll, chlorophyll fluorescence, and gaseous exchange, leading to higher biomass production. Functional genome analysis of the two most efficiently well-performing PGPR, Kosakonia cowanii HypNH10 and Rahnella variigena HypNH18, revealed several putative candidates, supporting their multifaceted plant growth-promoting attributes characterized in an *in vitro* assay, as well as their exorbitant plant growth-promoting performance observed in the greenhouse experiment. The pan-genomic study delivered insights into the several unique strain-specific functional genes contributing to root colonization, adaptation to plant immunity, and multifaceted plant-beneficial attributes. In summary, based on the *in vitro* and *in planta* plant growth-promoting assay coupled with comprehensive genome mining for unique PGPTs and secondary metabolite biosynthetic gene cluster analysis, here we recommended two *H. perforatum*-associated PGPR, *K. cowanii* HypNH10 and *R. variigena* HypNH18, as cogent bioinoculants for microbe-assisted cultivation of *H. perforatum* in the Indian western Himalaya. This study is the first pivotal step and a basis for future studies to develop microbe-assisted sustainable agricultural practices for industrial-scale cultivation of *H. perforatum*. However, to validate the impact of harnessed PGPR strains on *H. perforatum*’s agronomical traits and bioactive secondary metabolites, rigorous field trials are still required in the future.

## MATERIALS AND METHODS

### Rhizospheric soil collection and physicochemical characterization.

Rhizospheric soil samples were collected from naturally growing *H. perforatum* at the flowering stage (mid-June) from the sloppy meadows of Mural Danda peak and forest fields of Dhumrera village, Shimla, Himachal Pradesh, India. Samples were collected from three sampling sites (Fig. 1), and five plants with intact roots adhered with soil were gathered from each sampling site in zip-lock bags and kept in a portable ice-cooled box. Samples were carried to the laboratory as soon as possible and until processing were kept in a cold room (8 ± 1°C). For isolation of PGPR, large soil aggregates, including nonrhizospheric soil, were discarded from every plant, and only soil adhered with roots was collected and mixed to form a composite sample.

### Isolation and characterization of rhizobacteria.

Two types of enrichment media, modified Pikovskaya’s medium supplemented with insoluble tri-calcium phosphate and bromophenol blue dye and Ashby’s broth without any nitrogen (N) source, were used to obtain efficient P solubilizers and N fixers, respectively. Approximately 10 g from the composite pool of rhizospheric soil was suspended in 90 mL of sterile modified Pikovskaya’s and nitrogen-free Ashby’s medium in an Erlenmeyer flask and placed in a shaking incubator (120 rpm) at 20 ± 1°C for enrichment purposes. After 72 h of incubation, the aliquots from each medium were serially diluted up to 10-fold (10^−9^); then 100 μL of each dilution was spread onto Pikovskaya’s and Ashby’s glucose agar medium, respectively. They were incubated at 20 ± 1°C for 24 to 72 h and checked intermittently for any halozone formation, which indicates the presence of phosphate-solubilizing and N-fixing rhizobacteria in the respective media. All these procedures were performed in a class II, type A2 biological safety cabinet (Thermo Scientific, USA). The pure isolates were preserved in glycerol stocks (40% vol/vol) and stored for prolonged use at −80°C.

### Assessment of *in vitro* plant growth-promoting traits and screening of putative isolates for the *in planta* plant growth-promotion assay.

Diverse plant growth-promoting activities such as macronutrient (NPK) mineralization, siderophore and indole-3-acetic acid (IAA) production, including 1-aminocyclopropane-1-carboxylate deaminase (ACCD) activity, were used to screen the PGPR isolates using previously described methods (72–74). Additionally, based on *in vitro* quantitative plant growth-promoting traits of rhizobacterial isolates, a multivariate principal-component analysis (PCA) was performed (PAST4) for screening the statistically significant putative PGPR strains for further plant assays.

### Molecular identification and phylogenetic characterization of putative PGPR isolates.

The bacterial lysate was prepared through heat denaturation inside a thermal cycler (Applied Biosystems) as per the standard program, and DNA was quantified with a NanoDrop device (Thermo Fisher Scientific, USA), followed by storage at −20°C until further use. 16S rRNA-based preliminary identification and analysis of evolutionary relationships among isolates were performed as previously described (75). Aquifex pyrophilus strain Kol5a was used as an outgroup organism.

### Plant materials, experimental design, and conditions for accessing the efficacy of bioinoculants in *in planta* plant growth-promotion under poly greenhouse conditions.

The experiment was executed in a poly greenhouse under natural photoperiodic conditions (16 h light/8 h dark), with a 22 ± 2°C average mean daily temperature and 50 to 60% relative humidity (RH). The trial was arranged in a completely randomized design with 15 replications per treatment. Each pot was planted with a single plant seedling, and eight different treatments consisting of eight putative PGPR were set up. Additionally, uninoculated control (mock) sets of pots were maintained to replicate the effect of the control condition (Table S6). *H. perforatum* seeds (cultivar [cv.] IHBT/HP-1) were sown before the frost (mid-October) by placing them in rows (30 cm away from each other) in a nursery bed composed of farm yard manure, sand, and soil (1:1:1) and then covering them with a thin layer of sand. *H. perforatum* seedlings (60-days-old) with an average shoot and root length of 1.38 ± 0.30 and 4.31 ± 1.37 cm, respectively (Fig. S9), were used for the *in planta* plant growth-promoting assay.

### Preparation of potting mixture and bioinoculants.

The soil used for the potting mixture was collected from the experimental fields of CSIR-Institute of Himalayan Bioresource Technology, Palampur, Himachal Pradesh, India. The soil was air-dried, sieved with a 2-mm mesh, and mixed thoroughly with cocopeat, sand, vermiculite, and perlite (1:2:1:1:1) to prepare a potting mixture. The potting mixture was sterilized twice by autoclaving at 121°C for 1 h at 24 h intervals. The earthen pots (diameter, 15 cm; capacity, 3 kg) were filled with the sterile potting mixture and then irrigated with sterile distilled water (SDW) for a week before the transplantation of seedlings. Isolates were grown in a sterile nutrient broth medium in an incubator shaker (20 ± 1°C; 120 rpm) for 48 to 72 h. Afterward, the cultures were centrifuged at 6000 × *g* to settle the cells, followed by resuspension in sterile normal saline (0.9%) by keeping the final concentration of cells at about 1 × 10^8^ CFU mL^−1^ (optical density at 600 nm [OD_600_], 1) (76).

### Bioinoculation procedures.

The 60-day-old *H. perforatum* seedlings were uprooted carefully with intact roots and washed with tap water to remove all unwanted adhered soil. The soil-free seedlings were washed thrice with sterile normal saline (0.9%) to avoid cross-contamination with the previously associated rhizomicrobiome. Washed seedlings devoid of microflora were dipped into the respective bacterial cell suspensions (1 × 10^8^ CFU mL^−1^) for 15 min and allowed to dry for 30 s, followed by transplantation into the corresponding pots. The mock-inoculated seedlings were immersed in SDW and transplanted. After transplantation, 10 mL of the respective bacterial inoculum was poured into every pot close to the roots, whereas for the mock-inoculated pots, 10 mL SDW was used. Additionally, a second inoculation (booster) was delivered 15 days after the first inoculation by pouring another 10 mL of corresponding bacterial inoculums (1 × 10^8^ CFU mL^−1^) in the vicinity of roots to ensure PGPR colonization in the newly emerging roots and to fasten bacterial growth (76). Watering was suspended for 24 h before and after inoculation procedures to preclude washing. Thereafter, seedlings were watered two times a week with SDW.

### Assessment of agronomical traits.

Destructive harvesting was carried out at two intervals, i.e., 45 and 90 days after booster treatment, to assess any changes in plant growth and biomass accumulation. The plant growth-contributing attributes such as shoot and root length (cm), root volume (mL) (77), below- and above-ground dry weight (mg DW), and number of branches per plant (number plant^−1^) were recorded.

### Estimation of leaf photosynthetic pigments, chlorophyll fluorescence, and gaseous exchange.

The photosynthetic pigments, *viz.*, Chl_a_ and Chl_b_, including carotenoids, were estimated as described earlier by Raj et al. (76) at 90 days after booster treatment by collecting fully expended healthy leaves. Additionally, the fluorescence characteristics of chlorophyll (Chl) were evaluated *in vivo* with a portable pulse amplitude modulation fluorometer (Junior PAM, Walz, Effeltrich, Germany) using Wincontrol-3 software. Plants were covered with a black cloth for 30 min, and fully expanded leaves were used for the measurement. Afterward, when reaction centers were open, the maximum photochemical quantum efficiency of photosystem II (F_v_/F_m_) was determined, where F_v_ is the maximum variable fluorescence (F_m_ − F_0_), and F_0_ and F_m_ are the minimum and maximum Chl fluorescence yield in the dark-adapted state, respectively. Minimal fluorescence (F_0_) was determined by the leaf excitation with a weak LED beam, while maximum fluorescence (F_m_) was evaluated after exposing leaves to a 600-ms pulse of saturating actinic white light. After dark adaptation, light (420 μmol photons m^−2^ s^−1^) was turned on, and a saturating pulse (8,000 μmol photons m^−2^ s^−1^) was administered at 60-s intervals for 10 min to determine the maximum (F_m_′) and minimum (F_o_′) intensity of fluorescence in the light-adapted state. A light dependence curve was established with the application of a series of nine saturating pulses, with rising actinic irradiance ranging from 0 to 820 μmol photons m^−2^ s^−1^ to determine the electron transport rate (ETR = ΦPSII × PAR × 0.5 × leaf absorptivity coefficient ([0.85]), photochemical quenching (qP = [F_m′_ − F_s_]/]F_m′_ − F_0′_]), and PSII operating efficiency (ΦPSII = [F_m′_ − F_s′_]/F_m′_) (60). Additionally, the leaf gaseous exchange-related attributes were recorded with a portable infrared gas analyzer (IRGA) having a portable leaf chamber of 2 cm by 3 cm (6 cm^2^) and a red-blue LED light source (LICOR-6400 XT, LICOR Biosciences, Lincoln, NE, USA) (76). Three individual plants, the same used for Chl fluorescence, were selected per treatment for the measurement. Pots were irrigated with SDW before measurements, and the parameters were measured every day from 9:00 am to 11:00 am with leaves by placing them inside the chamber to cover almost 40% of the chamber and touching the thermocouple from the underside. Chamber conditions were set as a CO_2_ concentration of 400 mmol mol^−1^ provided by 12 g CO_2_ cylinder cartridges (LI-COR Bioscience) using a CO_2_ mixer with a flow rate of 500 μmol s^−1^ and RH of 60 to 65%. The block temperature of the chamber was set at 20°C, LED light intensity was set at 800 μmol m^−2^ s^−1^, and the average leaf area was given as 2.4 cm^2^.

### Genome assembly, phylogenomics, comparative genomics, functional annotation of plant growth-promoting traits, and analysis of bacterial secondary metabolite gene clusters.

The genomic DNA of the two best-performing PGPR isolates, *viz.*, Kosakonia cowanii HypNH10 and Rahnella variigena HypNH18, was extracted using a HiPurA bacterial genomic DNA purification kit (Himedia, India) following the manufacturer’s instructions. After that, the DNA was sequenced using the Illumina MiSeq platform. The raw reads for HypNH10 were assembled using the Unicycler assembler v0.5.0 (78), while the assembly of HypNH18 was done using the SPAdes de novo assembler (79). The assembled genomic sequences of both strains were submitted to NCBI and annotated using the Prokaryotic Genome Annotation Pipeline v5.2 and prokka v2.14.6 (80, 81). The assembly files of both genomes were uploaded to Type Strain Genome Server to find the nearest relatives of the isolates (82). Thereafter, the pan-genome analysis of strains HypNH10 and HypNH18 with their closely related strains was done using the Roary v3.13.0 tool with a cutoff identity of 90% (83). The unique genes in both strains were also annotated by performing a BLASTp search against the PGPT protein reference sequence database using the DIAMOND tool (84). The difference in the genes between our strains and their related strains was determined using the “query_pan_genome” command. The unique genes present in strains HypNH10 and HypNH18 were then functionally annotated for the plant growth-promoting trait (PGPT) ontology using a novel PGPT-Pred tool available on the web platform for plant-associated bacteria, PLaBAse (85, 86). Further, a circular genome map was prepared by taking strains HypNH10 and HypNH18 as reference strains against their related strains using the BLAST Ring Image Generator (BRIG) v0.95 (87). The unique functional genes related to PGPTs present in the strains were plotted in the outermost ring. The secondary metabolite biosynthesis gene clusters (BGCs) in the genomes were predicted using the web-based server AntiSMASH v6.0 (https://antismash.secondarymetabolites.org/#!/start) (88).

### Statistical analysis.

All measurements were done in triplicates (*n* = 3); after that, data were processed through a one-way analysis of variance (ANOVA) followed by *post hoc* comparisons with Duncan’s multiple-range test (*P* < 0.05) (Statistics v26; SPSS, IBM Company, USA).

### Data availability.

The 16S rRNA nucleotide sequences of eight statistically significant multifaceted PGPR associated with *Hypericum perforatum* L. Enterobacter huaxiensis HypNH1, Flavobacterium acidificum HypNH2, Kocuria palustris HypNH4, Rahnella woolbedingensis HypNH8, Kosakonia cowanii HypNH10, Staphylococcus edaphicus HypNH14, Curtobacterium albidum HypNH15, and Rahnella variigena HypNH18 generated during the study were submitted to NCBI GenBank/DDBJ/EMBL under accession numbers OP848236 to OP848243, while the draft genomes of two best-performing PGPR, Kosakonia cowanii HypNH10 and Rahnella variigena HypNH18 were submitted under accession numbers JAOTIU000000000 and JAOTIV000000000.
