# Supplementary material for: Comparative Genomics and Physiological Investigations Supported Multifaceted Plant Growth-Promoting Activities in Two Hypericum perforatum L.-Associated Plant Growth-Promoting Rhizobacteria for Microbe-Assisted Cultivation
Source: Microbiol Spectr. 2023 May 18;11(3):e00607-23. doi: 10.1128/spectrum.00607-23 (PMC10269543; doi:10.1128/spectrum.00607-23)
Supplement: Supplemental file 1 — Supplemental material. Download spectrum.00607-23-s0001.pdf, PDF file, 3.0 MB [file spectrum.00607-23-s0001.pdf]

Supplementary Information

Title: Comparative genomics and physiological investigations supported multifaceted plant growth-promoting activities in two *Hypericum perforatum* L.-associated PGPR for microbe-assisted cultivation

Authors: Yog Raj[1,3], Anil Kumar[2,3], Sareeka Kumari[2,3], Rakshak Kumar[2,3,*], and Rakesh Kumar[1,3,*]

Affiliations: [1]Agrotechnology Division, CSIR-Institute of Himalayan Bioresource Technology, Palampur–176 061, India.

[2]High Altitude Microbiology Laboratory, Biotechnology Division, CSIR-Institute of Himalayan Bioresource Technology, Palampur–176 061, India.

[3]Academy of Scientific and Innovative Research (AcSIR), Ghaziabad–201 002, India

***Corresponding Authors**

1. Dr Rakesh Kumar

Email: rakeshkumar@ihbt.res.in; rakeshkrana@yahoo.com

Tel No. +91 1894–233339; Fax No. +91 1894–230433

2. Dr Rakshak Kumar

Email: rakshak@ihbt.res.in; rakshakacharya@gmail.com

Tel no. +91 1894–233339; Fax No. +91 1894–230433

The following supporting information is available for this article:

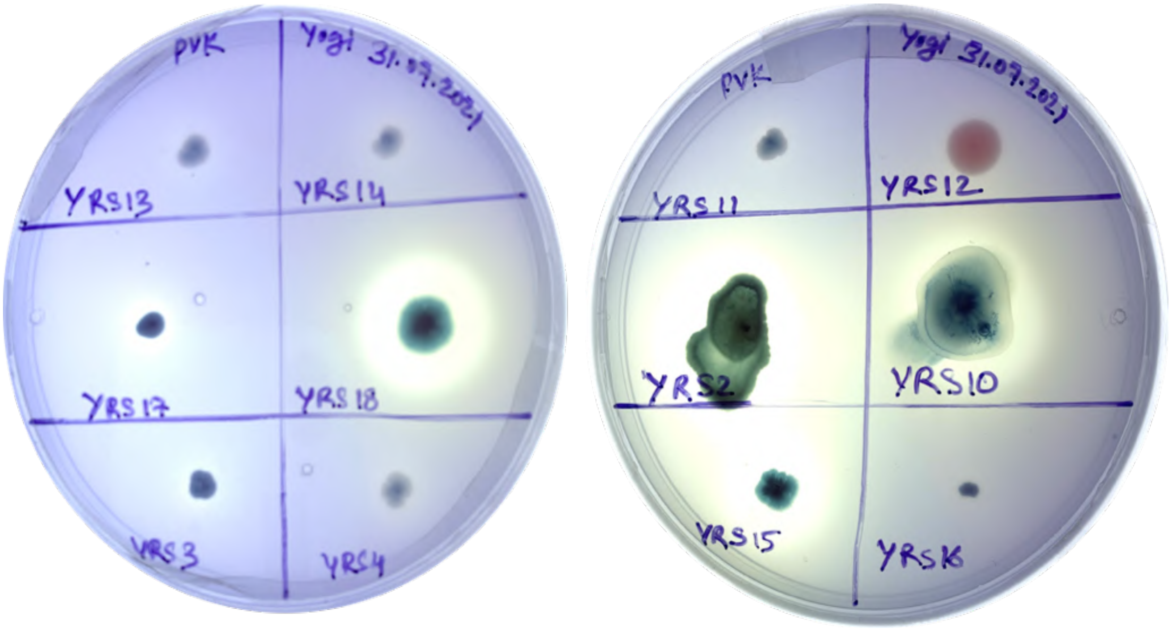

Fig. S1. Phosphate solubilization by *Hypericum perforatum* L.-associated rhizobacterial isolates in Pikovaskya's plate assay

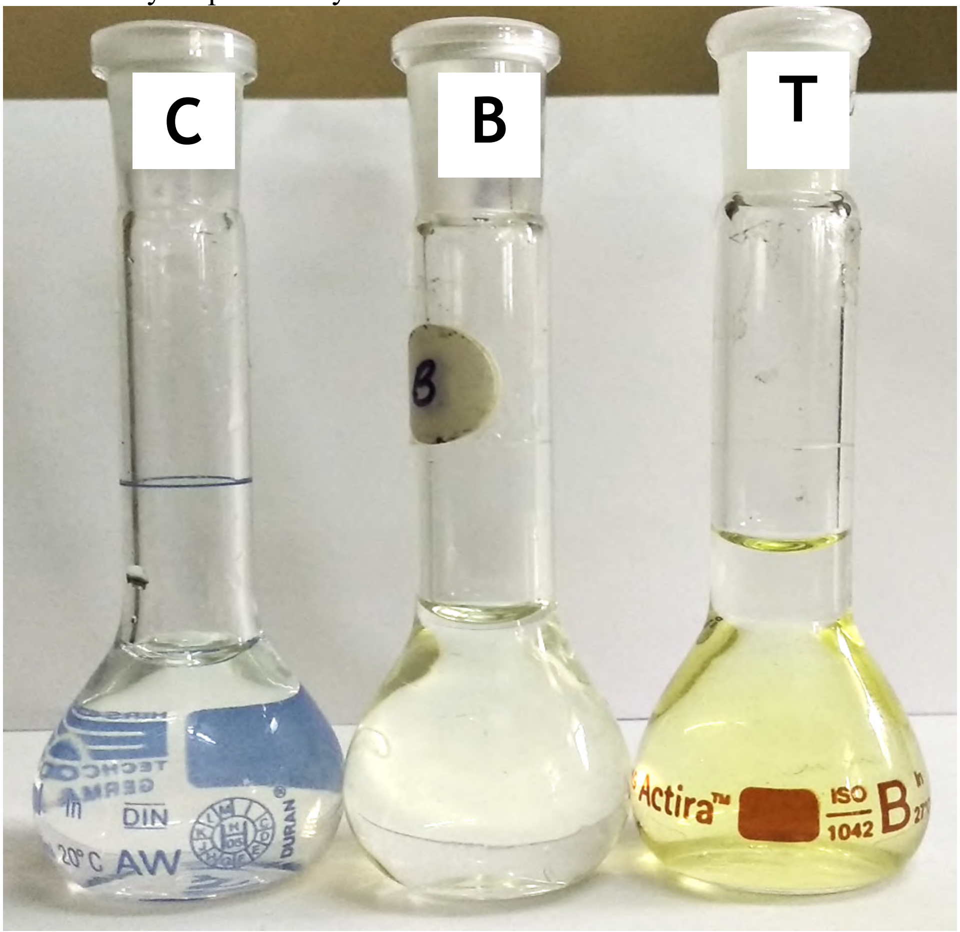

Fig. S2. Quantitative assessment of phosphate solubilization by *H. perforatum*-associated rhizobacterial isolates. T: test; B: blank; C: control

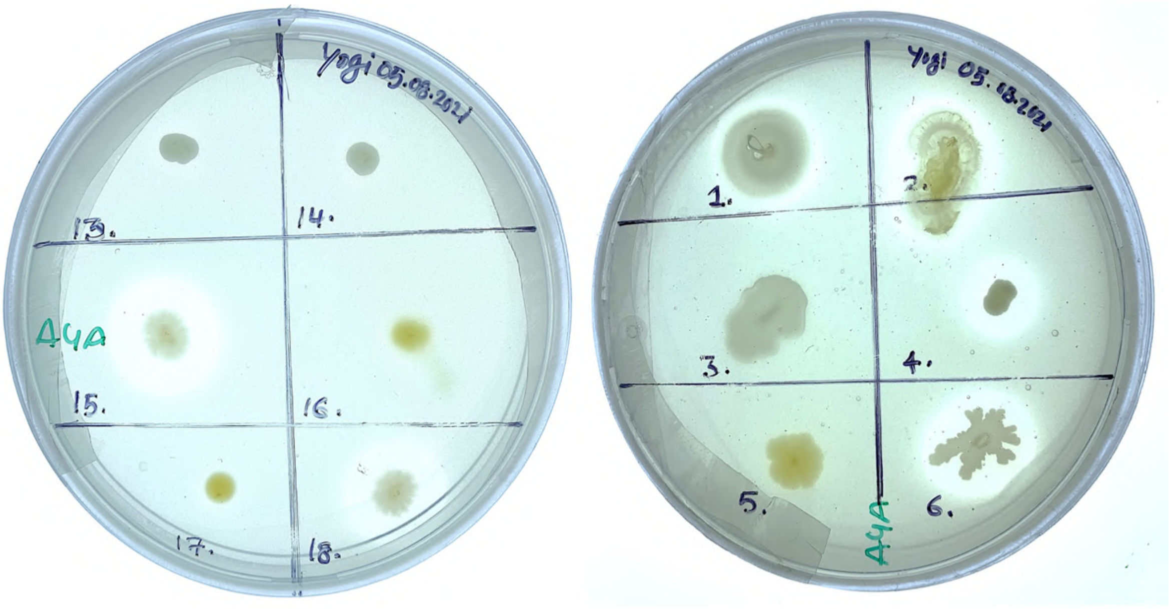

Fig. S3. Atmospheric nitrogen fixation by *H. perforatum*-associated rhizobacterial isolates on Ashby's glucose agar plate assay

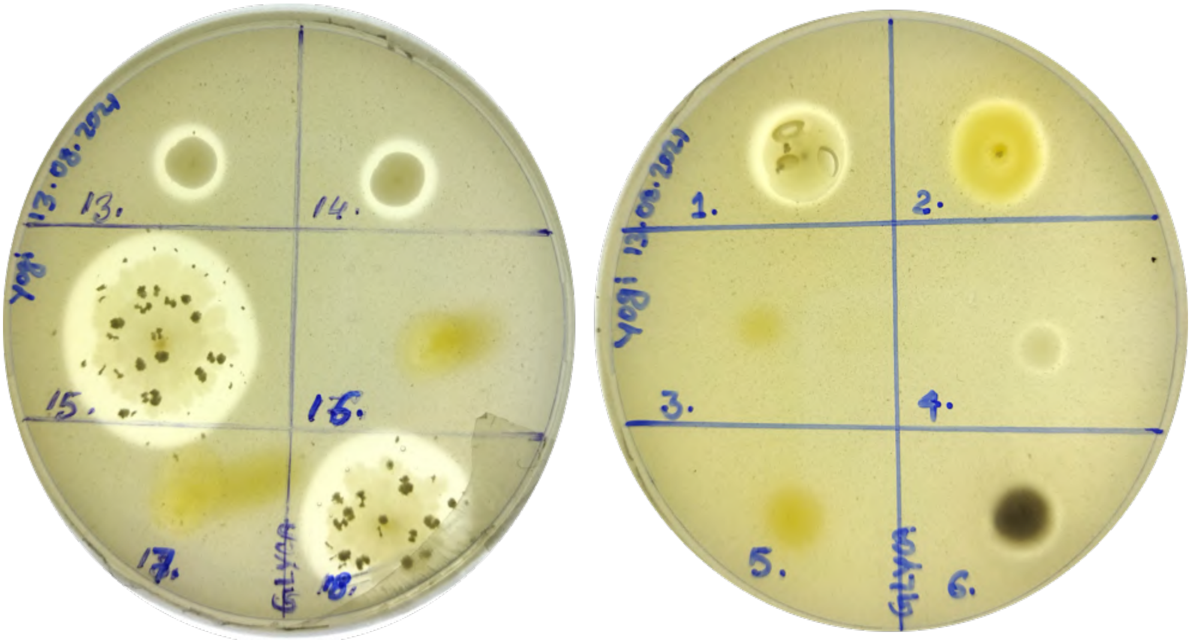

Fig. S4. Potassium mobilization by *H. perforatum*-associated rhizobacterial isolates on Glucose yeast agar plate assay

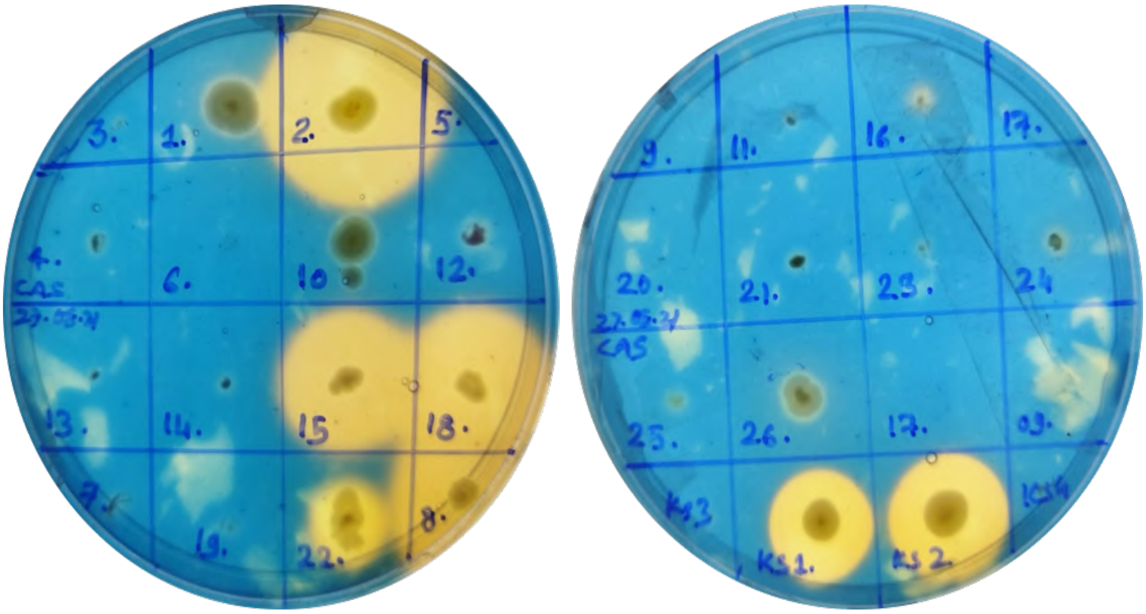

Fig. S5. Qualitative assay for siderophore production by *H. perforatum*-associated rhizobacterial isolates on Chrome azurol S media

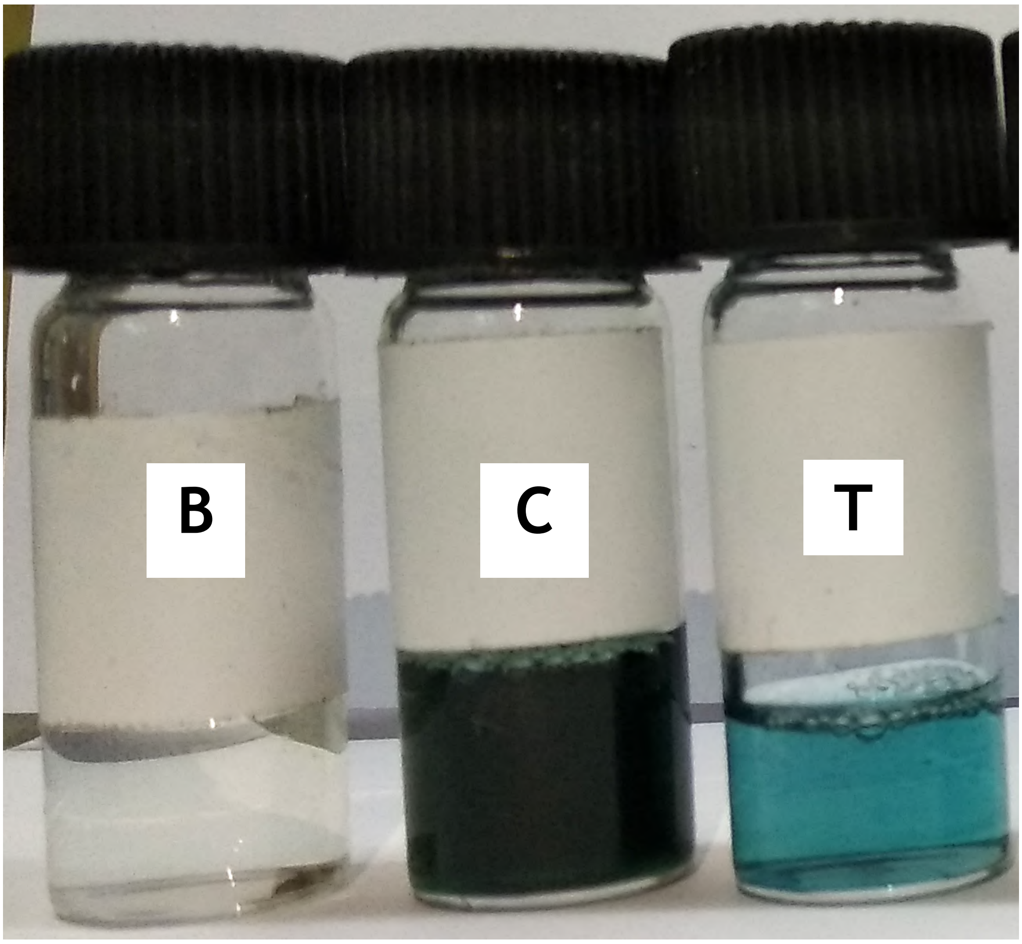

Fig. S6. Quantitative assessment of siderophore production by *H. perforatum*-associated rhizobacterial isolates. T: test; B: blank; C: control

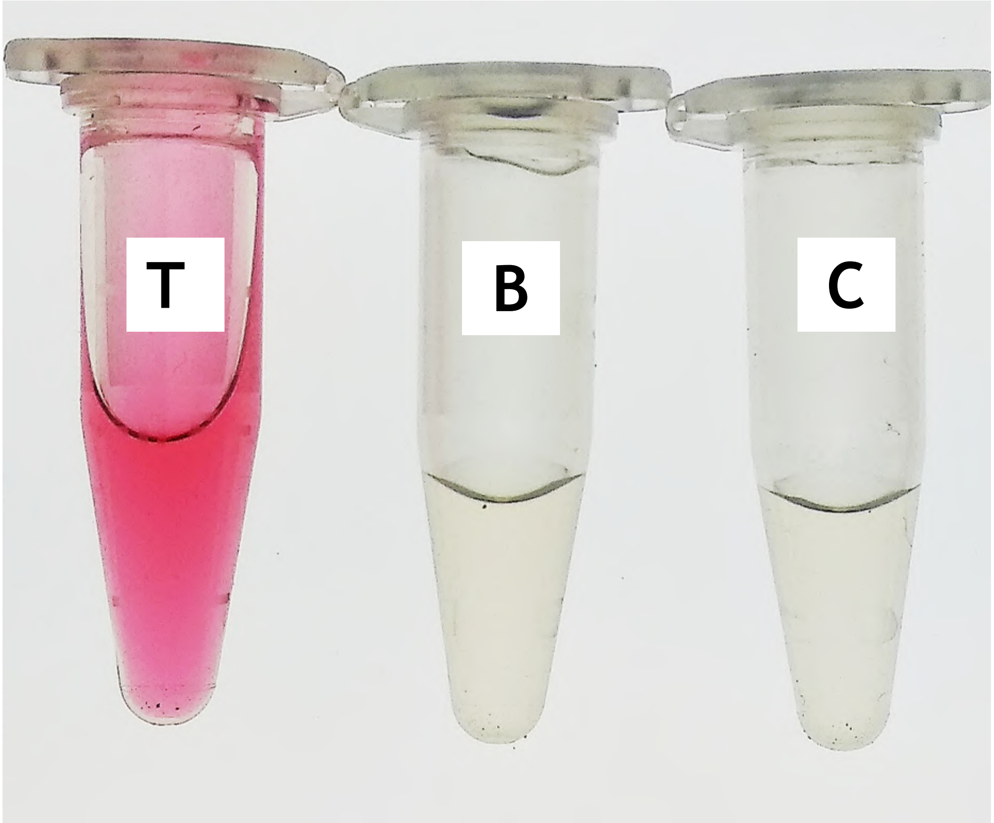

Fig. S7. Quantitative assessment of indole-3-acetic acid (IAA) production by *H. perforatum*-associated rhizobacterial isolates in the presence of L-tryptophan. T: test; B: blank; C: control

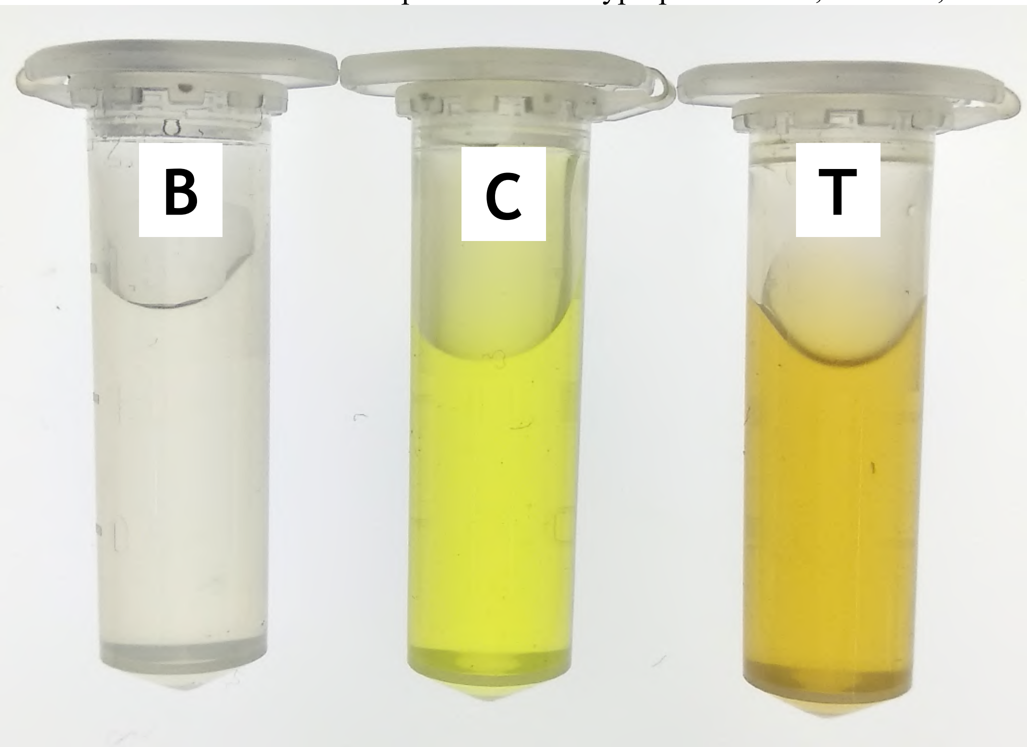

Fig. S8. Quantitative assessment of ACCD (1-aminocyclopropane-1-carboxylate deaminase) activity by *H.perforatum*-associated rhizobacterial isolates. T: test; B: blank; C: control

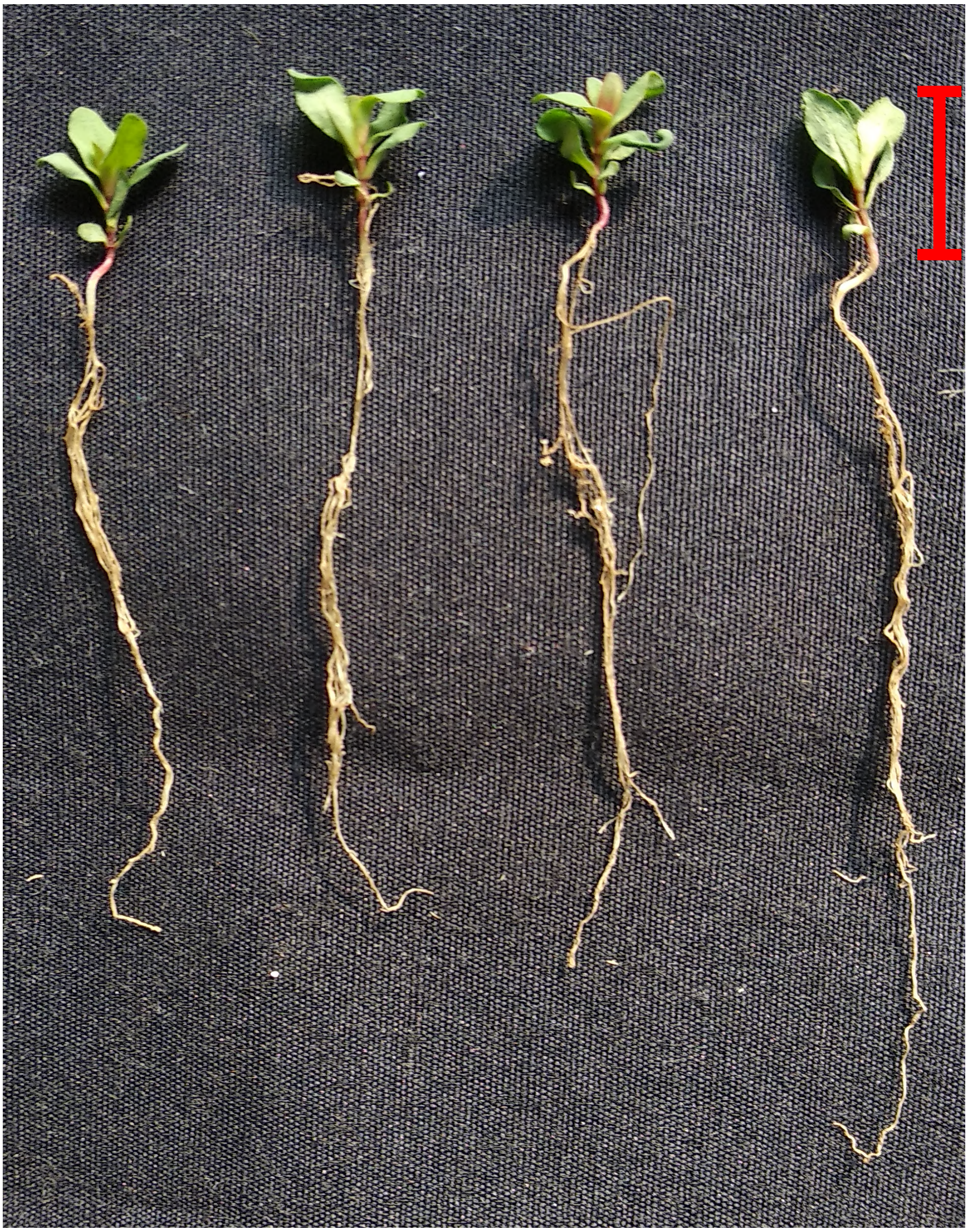

Fig. S9. Sixty days old seedlings of *H. perforatum* used for bioinoculation procedures and *in-planta* plant growth-promotion assay under a poly greenhouse conditions

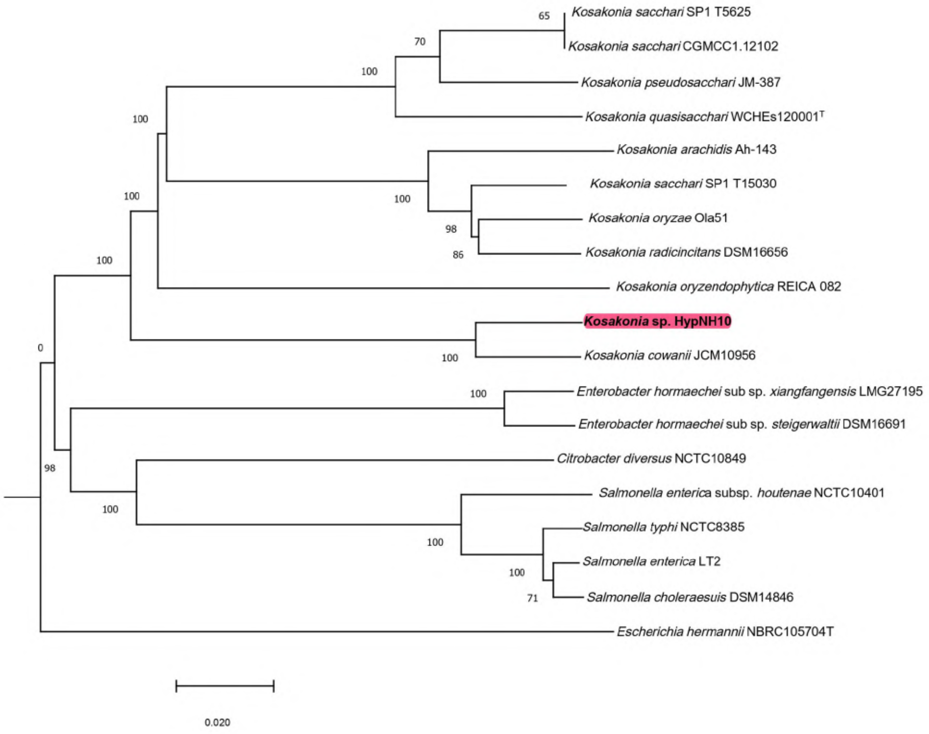

b) Phylogenomic tree of strain *Kosakonia cowanii* HypNH10

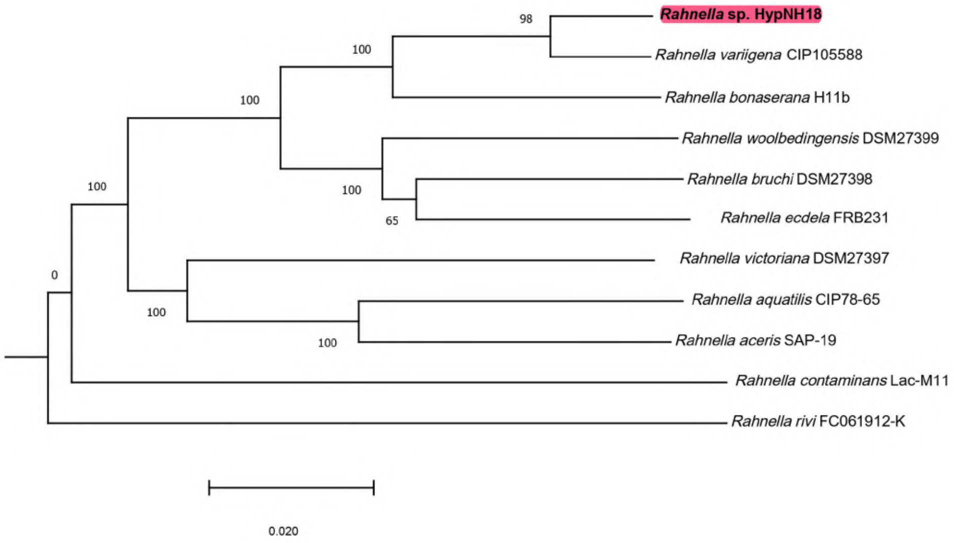

b) Phylogenomic tree of strain *Rahnella variigena* HypNH18

Fig. S10. Type Strain Genome Server based phylogenomic tree of studied strains. (a) Phylogenomic tree of strain HypNH10. The tree showed the clustering of strain HypNH10 with *Kosakonia cowanii* JCM10956. (b) Phylogenomic tree of strain HypNH18. The tree showed the clustering of strain HypNH10 with *Rahnella variigena* CIP105588

a) *Kosakonia cowanii* HypNH10

Overview 1.1 1.2 1.3 1.4 5.1 6.1 10.1

Identified secondary metabolite regions using strictness 'relaxed'

Region	Type	From	To	Most similar known cluster		Similarity
Region 1.1	terpene	336,334	359,912	carotenoid	Terpene	100%
Region 1.2	ladderane	462,611	505,172			
Region 1.3	thiopeptide	560,728	587,018	O-antigen	Saccharide	14%
Region 1.4	NRPS	911,334	955,221	turnerbactin	NRP	30%
Region 5.1	redox-cofactor	264,567	286,726	lankacidin C	NRP + Polyketide	13%
Region 6.1	NRPS	65,389	138,091	teicoplanin	NRP:Glycopeptide	3%
Region 10.1	RiPP-like	86,819	95,034			

b) *Rahnella variigena* HypNH18

Overview 1.1 3.1 4.1 7.1 47.1

Identified secondary metabolite regions using strictness 'relaxed'

Region	Type	From	To	Most similar known cluster		Similarity
Region 1.1	RRE-containing	116,791	137,078	lankacidin C	NRP + Polyketide	13%
Region 3.1	betalactone	114,748	140,117			
Region 4.1	siderophore	178,091	190,475	desferrioxamine E	Other	100%
Region 7.1	hserlactone	78,684	99,304			
Region 47.1	arylpolyene	1	29,481	aryl polyenes	Other	61%

Fig. S11. AntiSMASH analyses showing bacterial secondary metabolite biosynthetic gene clusters (BGCs) and their genomic location in two best performing PGPR (a) *Kosakonia cowanii* HypNH10 and (b) *Rahnella variigena* HypNH18.

Table S1. Colony morphology of *H. perforatum*-associated rhizobacterial isolates

Sr no	Isolate code	Shape	Size	Colour	Texture	Elevation	Margin	Transparency	Dry/Wet
1	HypNH1	Circular	M	W	SS	Flat	Irregular	Opaque	Wet
2	HypNH2	Irregular	M	LY	SS	Convex	Irregular	opaque	wet
3	HypNH3	Circular	M	LY	SS	Low convex	Fixed circular	Opaque	Wet
4	HypNH4	Circular	S	W	SS	Convex	Fixed circular	Opaque	Wet
5	HypNH5	Circular	S	LY	SS	Low convex	Fixed circular	Opaque	Wet
6	HypNH6	Rhizoid	L	W	RD	Flat	Irregular	Translucent	Dry
7	HypNH7	Rhizoid	S	W	RD	Flat	Irregular	Opaque	Dry
8	HypNH8	Circular	S	W	SS	Convex	Fixed circular	Opaque	Wet
9	HypNH9	Irregular	L	W	SS	Convex	Irregular	Translucent	Wet
10	HypNH10	Irregular	L	W	RD	Low convex	Irregular	Translucent	Dry
11	HypNH11	Circular	S	W	SS	Convex	Fixed circular	Translucent	Wet
12	HypNH12	Circular	S	P	SS	Low convex	Irregular	Opaque	Wet
13	HypNH13	Circular	M	W	SS	Convex	Fixed circular	Opaque	Wet
14	HypNH14	Circular	S	W	SS	Convex	Fixed circular	Opaque	Wet
15	HypNH15	Circular	L	W	RS	Flat	Irregular	Opaque	Wet
16	HypNH16	Circular	S	W	SS	Low convex	Fixed circular	Opaque	Wet
17	HypNH17	Circular	M	W	SS	Low convex	Fixed circular	Translucent	Wet
18	HypNH18	Circular	M	W	SS	Low convex	Fixed circular	Translucent	Wet
19	HypNH19	Circular	S	LY	SS	Convex	Fixed circular	Opaque	Wet
20	HypNH20	Circular	S	LY	SS	Convex	Fixed circular	Opaque	Wet
21	HypNH21	Circular	M	W	SS	Convex	Fixed circular	Opaque	Wet
22	HypNH22	Circular	L	W	RS	Flat	Fixed circular	Opaque	Wet
23	HypNH23	Circular	L	W	SS	Convex	Fixed circular	Opaque	Wet
24	HypNH24	Circular	M	W	SS	Low convex	Fixed circular	Opaque	Wet
25	HypNH25	Circular	S	W	SS	Low convex	Fixed circular	Opaque	Wet
26	HypNH26	Circular	M	W	RS	Flat	Fixed circular	Opaque	Dry
27	HypNH27	Irregular	S	W	RS	Flat	Irregular	Opaque	Dry
28	HypNH28	Circular	M	W	SS	Low convex	Fixed circular	Opaque	Wet
29	HypNH29	Circular	S	LY	SS	Flat	Fixed circular	Opaque	Wet
30	HypNH30	Irregular	L	W	RS	Convex	Irregular	Opaque	Dry
31	HypNH31	Circular	S	W	SS	Flat	Fixed circular	Opaque	Wet

L: large; M: medium; S: small; W: white; LY: light yellow; P: pink; SS: smooth shiny; RD: rough dull

Table S2. Phosphate solubilizing index (PSI) shown by *H. perforatum*-associated rhizobacterial isolates

Sr no	Isolate code	Halozone diameter(cm)	Colony diameter (cm)	Zone of hydrolysis (cm)	PSI
1	HypNH1	0.97	0.53	0.44	2.36
2	HypNH2	2.20	1.70	0.50	2.99
3	HypNH3	0.80	0.70	0.10	1.84
4	HypNH4	1.32	1.30	0.02	2.32
5	HypNH5	0.45	0.35	0.10	1.64
6	HypNH6	ND	0.75	ND	ND
7	HypNH7	0.90	0.80	0.10	1.93
8	HypNH8	0.35	0.25	0.10	1.65
9	HypNH9	NZ	5.30	ND	ND
10	HypNH10	2.20	1.50	0.70	2.97
11	HypNH11	0.40	0.35	0.05	1.49
12	HypNH12	0.60	0.50	0.10	1.70
13	HypNH13	1.37	1.35	0.02	2.36
14	HypNH14	1.32	1.30	0.02	2.32
15	HypNH15	1.70	0.80	0.90	2.93
16	HypNH16	0.30	0.25	0.05	1.45
17	HypNH17	0.65	0.60	0.05	1.68
18	HypNH18	2.60	1.45	1.15	3.24
19	HypNH19	0.45	0.40	0.05	1.53
20	HypNH20	0.60	0.40	0.20	1.90
21	HypNH21	NZ	0.80	ND	ND
22	HypNH22	1.50	1.20	0.30	2.45
23	HypNH23	NZ	0.90	ND	ND
24	HypNH24	NZ	0.70	ND	ND
25	HypNH25	NZ	0.45	ND	ND
26	HypNH26	0.70	0.60	0.10	1.77
27	HypNH27	NZ	0.58	ND	ND
28	HypNH28	NZ	0.23	ND	ND
29	HypNH29	NZ	0.78	ND	ND
30	HypNH30	NZ	0.89	ND	ND
31	HypNH31	NZ	0.48	ND	ND

Results are presented as means of three replicates (*n=3*); PSI: phosphate solubilizing index (CD+HD/CD); NZ: no zone; ND: not determine

Table S3. Nitrogen fixation index (NFI) shown by *H. perforatum*-associated rhizobacterial isolates

Sr no	Isolate code	Halozone diameter(cm)	Colony diameter (cm)	Zone of hydrolysis (cm)	NFI
1	HypNH1	1.52	1.5	0.02	2.51
2	HypNH2	1.85	1.45	0.40	2.73
3	HypNH3	NZ	1.35	ND	ND
4	HypNH4	1.60	0.80	0.80	2.80
5	HypNH5	NZ	0.70	ND	ND
6	HypNH6	1.90	1.20	0.70	2.78
7	HypNH7	1.23	1.20	0.03	2.23
8	HypNH8	0.75	0.70	0.05	1.89
9	HypNH9	NZ	3.50	ND	ND
10	HypNH10	2.50	2.20	0.30	3.34
11	HypNH11	0.53	0.50	0.03	1.56
12	HypNH12	0.72	0.70	0.02	1.73
13	HypNH13	0.69	0.60	0.09	1.75
14	HypNH14	0.53	0.50	0.03	1.56
15	HypNH15	2.10	0.90	1.20	3.23
16	HypNH16	NZ	0.80	ND	ND
17	HypNH17	0.62	0.60	0.02	1.63
18	HypNH18	1.80	0.80	1.00	3.05
19	HypNH19	NZ	0.40	ND	ND
20	HypNH20	NZ	0.40	ND	ND
21	HypNH21	NZ	0.80	ND	ND
22	HypNH22	NZ	1.20	ND	ND
23	HypNH23	NZ	0.90	ND	ND
24	HypNH24	NZ	0.70	ND	ND
25	HypNH25	NZ	0.45	ND	ND
26	HypNH26	NZ	0.60	ND	ND
27	HypNH27	NZ	NG	ND	ND
28	HypNH28	NZ	NG	ND	ND
29	HypNH29	NZ	NG	ND	ND
30	HypNH30	NZ	NG	ND	ND
31	HypNH31	NZ	NG	ND	ND

Results are presented as means of three replicates (*n=3*); NFI: nitrogen fixation index (CD+HD/CD); NZ: no zone; NG: no growth ND: not determined

Table S4. Potassium mobilizing index (KMI) shown by *H. perforatum*-associated rhizobacterial isolates

Sr No	Isolate code	Halozone diameter(cm)	Colony diameter (cm)	Zone of hydrolysis (cm)	KMI
1	HypNH1	1.90	1.60	0.30	2.79
2	HypNH2	1.85	1.70	0.15	2.79
3	HypNH3	NZ	0.90	ND	ND
4	HypNH4	1.80	1.10	0.70	2.74
5	HypNH5	NZ	1.10	ND	ND
6	HypNH6	2.37	2.35	0.35	1.50
7	HypNH7	1.65	1.60	0.05	2.63
8	HypNH8	0.85	0.73	0.12	1.89
9	HypNH9	NZ	1.63	ND	ND
10	HypNH10	1.90	1.70	0.20	2.82
11	HypNH11	0.73	0.55	0.18	1.88
12	HypNH12	NZ	0.70	ND	ND
13	HypNH13	0.90	0.70	0.20	1.99
14	HypNH14	0.85	0.70	0.15	1.91
15	HypNH15	2.40	1.95	0.45	3.50
16	HypNH16	NZ	1.00	ND	ND
17	HypNH17	NZ	1.00	ND	ND
18	HypNH18	2.30	1.60	0.70	3.18
19	HypNH19	0.90	0.80	0.10	1.93
20	HypNH20	NZ	0.50	ND	ND
21	HypNH21	NZ	0.80	ND	ND
22	HypNH22	NZ	0.70	ND	ND
23	HypNH23	NZ	0.34	ND	ND
24	HypNH24	NZ	0.45	ND	ND
25	HypNH25	NZ	0.25	ND	ND
26	HypNH26	NZ	0.40	ND	ND
27	HypNH27	NZ	NG	ND	ND
28	HypNH28	NZ	0.38	ND	ND
29	HypNH29	NZ	0.44	ND	ND
30	HypNH30	NZ	NG	ND	ND
31	HypNH31	NZ	NG	ND	ND

Results are presented as means of three replicates (*n=3*); KMI: potassium mobilizing index (CD+HD/CD); NZ: no zone; NG: no growth; ND: not determined

Table S5. Siderophore producing index (SPI) shown by *H. perforatum*-associated rhizobacterial isolates

Sr No	Isolate code	Holozone diameter(cm)	Colony diameter (cm)	Zone of hydrolysis (cm)	SPI
1	HypNH1	NZ	0.90	ND	ND
2	HypNH2	2.90	0.80	2.10	4.43
3	HypNH3	NZ	NG	ND	ND
4	HypNH4	NZ	0.30	ND	ND
5	HypNH5	NZ	NG	ND	ND
6	HypNH6	NZ	NG	ND	ND
7	HypNH7	NZ	NG	ND	ND
8	HypNH8	1.70	0.60	1.10	3.43
9	HypNH9	NZ	NG	ND	ND
10	HypNH10	NZ	0.65	ND	ND
11	HypNH11	NZ	0.25	ND	ND
12	HypNH12	NZ	0.50	ND	ND
13	HypNH13	NZ	0.20	ND	ND
14	HypNH14	NZ	0.20	ND	ND
15	HypNH15	2.10	0.60	1.50	4.10
16	HypNH16	0.4	0.20	0.20	2.20
17	HypNH17	NZ	NG	ND	ND
18	HypNH18	2.30	0.50	1.80	5.1
19	HypNH19	NZ	NG	ND	ND
20	HypNH20	NZ	NG	ND	ND
21	HypNH21	NZ	0.30	ND	ND
22	HypNH22	1.20	0.85	0.35	2.26
23	HypNH23	NZ	0.20	ND	ND
24	HypNH24	0.4	0.25	0.15	1.85
25	HypNH25	0.4	0.15	0.25	2.82
26	HypNH26	0.65	0.60	0.05	1.68
27	HypNH27	NZ	NG	ND	ND
28	HypNH28	NZ	NG	ND	ND
29	HypNH29	NZ	NG	ND	ND
30	HypNH30	NZ	NG	ND	ND
31	HypNH31	NZ	NG	ND	ND

Results are presented as means of three replicates (*n=3*); SPI: siderophore producing index (CD+HD/CD); NZ: no zone; NG: no growth; ND: not determined

Table S6. Quantitative plant growth-promoting attributes of *H. perforatum*-associated rhizobacterial isolates

Isolate codes	IAA (μg mL^{-1})	Phosphate solubilization (μg mL^{-1})	Siderophore producing unit (psu)	ACCD (nmol α-ketobutyrate mg^{-1} h^{-1})
HypNH1	84.89	39.77	84.01	481.45
HypNH2	63.72	56.43	87.29	447.81
HypNH3	0.59	34.43	90.89	0.00
HypNH4	67.24	53.10	93.86	901.05
HypNH5	3.59	33.43	84.54	103.60
HypNH6	10.53	37.77	0.00	0.00
HypNH7	20.88	39.43	0.00	0.00
HypNH8	32.09	71.43	78.40	1227.64
HypNH9	3.70	15.77	0.00	0.00
HypNH10	55.87	61.10	98.46	615.10
HypNH11	30.70	25.10	83.74	129.41
HypNH12	54.15	17.43	98.92	145.31
HypNH13	28.87	28.77	96.86	0.00
HypNH14	52.29	41.43	97.08	773.82
HypNH15	37.64	50.10	97.78	615.10
HypNH16	7.48	24.10	99.92	0.00
HypNH17	1.98	25.77	99.42	0.00
HypNH18	54.68	64.43	74.90	1296.42
HypNH19	2.69	27.43	0.00	0.00
HypNH20	14.77	38.10	97.21	0.00
HypNH21	5.02	28.43	63.12	141.80
HypNH22	4.31	41.10	98.12	0.00
HypNH23	6.81	49.10	82.27	0.00
HypNH24	10.79	24.77	99.04	192.08
HypNH25	28.04	26.77	97.16	0.00
HypNH26	3.25	37.43	88.70	282.77
HypNH27	0.00	0.00	0.00	0.00
HypNH28	0.00	0.00	0.00	0.00
HypNH29	0.00	0.00	0.00	0.00
HypNH30	0.00	0.00	0.00	0.00
HypNH31	0.00	0.00	0.00	192.08

Results are presented as means of three replicates (*n=3*) and further used for multivariate principle component analysis (PCA)

Table S7. Identification of eight putative PGPR strains based on the 16S rRNA gene high percentage (>98%) similarity with validly published strains on Eztaxon database

Sr No	Identified rhizobacteria	Top-hit taxon	Top-hit strain	Similarity (%)	NCBI accession number
1	*Enterobacter* sp. Hyp NH1	*Enterobacter huaxiensis*	090008	99.70	OP48236
2	*Flavobacterium* sp. HypNH2	*Flavobacterium acidificum*	LMG 8364	100.00	OP48237
3	*Kocuria* sp. HypNH4	*Kocuria palustris*	DSM 11925	99.71	OP48238
4	*Rahnella* sp. HypNH8	*Rahnella woolbedingensis*	DSM 27399	99.65	OP48239
5	*Kosakonia* sp. HypNH10	*Kosakonia cowanii*	JCM10956(T)	99.73	OP48240
6	*Staphylococcus* sp. HypNH14	*Staphylococcus edaphicus*	P5085	100.00	OP48241
7	*Curtobacterium* sp. HypNH15	*Curtobacterium albidum*	DSM 20512	98.41	OP48242
8	*Rahnella* sp. HypNH18	*Rahnella variigena*	NSDJ01000001	99.48	OP48243

Table S8. Details of treatments and abbreviations

Abbreviations	Details
T1	Sterile potting mixture without inoculation (Mock)
T2	Treatment with *Enterobacter huaxiensis* HypNH1 cells ($OD_{600,}$ 1) suspended in sterile 0.9% normal saline
T3	Treatment with *Flavobacterium acidificum* HypNH2 cells ($OD_{600,}$ 1) suspended in sterile 0.9% normal saline
T4	Treatment with *Kocuria palustris* HypNH4 cells ($OD_{600,}$ 1) suspended in sterile 0.9% normal saline
T5	Treatment with *Rahnella woolbedingensisi* HypNH8 cells ($OD_{600,}$ 1) suspended in sterile 0.9% normal saline
T6	Treatment with *Kosakonia cowanii* HypNH10 cells ($OD_{600,}$ 1) suspended in sterile 0.9% normal saline
T7	Treatment with *Staphylococcus edaphicus* HypNH14 cells ($OD_{600,}$ 1) suspended in sterile 0.9% normal saline
T8	Treatment with *Curtobacterium albidum* HypNH15 cells ($OD_{600,}$ 1) suspended in sterile 0.9% normal saline
T9	Inoculation with *Rahnella variigena* HypNH18 cells ($OD_{600,}$ 1) suspended in sterile 0.9% normal saline

Inoculation events: i) first inoculation at the time of transplanting and ii) second inoculation (booster) 15 days after first inoculation event

Table S9. Comparative genome characteristics of *Kosakonia cowanii* HypNH10 with complete/near-complete genomes of *K. cowanii.*

Attributes	*Kosakonia cowanii* HypNH10	*Kosakonia cowanii* JCM 10956=DSM 18146 stra	*Kosakonia cowanii strain* FBS 223	*Kosakonia cowanii* strain LT-1
Genome size (bp)	4.72	4.81	4.69	4.77
GC content (%)	55.00	56.20	56.20	56.12
Contigs	23	108	1	2
Total genes	4,470	4,608	4,421	4,462
CDS (Coding)	4,310	4,338	4,246	4,298
rRNAs	3	22	22	22
tRNAs	71	83	83	82
ncRNA	15	9	10	9
Pseudogenes	71	156	60	51
Genome coverage	200.0x	80x	453.94x	100x
Sequencing technology	Illumina MiSeq	PacBio	Oxford Nanopore GridION	Hybrid (Illumina HiSeq+PacBio)

Table S10. Comparative genome characteristics of *Rahnella variigena* HypNH18 with complete/near-complete genomes of *R. variigena*

Attributes	*Rahnella variigena* HypNH18	*R. variigena* Strain: CIP 105588	*R. variigena* Strain: VCR3
Genome size (bp)	5.32	5.05	5.39
GC content (%)	52.15	56.01	52.10
Contigs	104	66	8
Total genes	4,915	4,978	4,944
CDS (Coding)	4,798	4,823	4,772
rRNAs	4	4	12
tRNAs	39	68	69
ncRNA	9	8	10
Pseudo genes	65	75	81
Genome coverage	200.0x	80.0x	12x
Sequencing technology	Illumina MiSeq	Illumina NextSeq 500	Illumina

Table S11. Pan-genome analysis statistics

Attributes	*Kosakonia cowanii* HypNH10	*Rahnella variigena* HypNH18
Total genes	36506	21275
Cloud genes	27010	14701
Shell genes	9305	5077
Core genes	191	1497
